# Synthesis, Mesomorphism and the Optical Properties of Alkyl-deuterated Nematogenic 4-[(2,6-Difluorophenyl)ethynyl]biphenyls

**DOI:** 10.3390/ma14164653

**Published:** 2021-08-18

**Authors:** Jakub Herman, Piotr Harmata, Michał Czerwiński, Olga Strzeżysz, Marta Pytlarczyk, Monika Zając, Przemysław Kula

**Affiliations:** Faculty of Advanced Technologies and Chemistry, Military University of Technology, 2 gen. S. Kaliskiego St., 00-908 Warsaw, Poland; jakub.herman@wat.edu.pl (J.H.); piotr.harmata@wat.edu.pl (P.H.); michal.czerwinski@wat.edu.pl (M.C.); olga.strzezysz@wat.edu.pl (O.S.); marta.pytlarczyk@wat.edu.pl (M.P.); monika.zajac@wat.edu.pl (M.Z.)

**Keywords:** infrared, flow chemistry, deuterium addition, nematic

## Abstract

The synthesis and characterization of new deuterated liquid crystal (LC) compounds based on phenyl tolane core is described in this paper. The work presents an alternative molecular approach to the conventional LC design. Correlations between molecular structure and mesomorphic and optical properties for compounds which are alkyl-hydrogen terminated and alkyl-deuterium, have been drawn. The compounds are characterized by mass spectrometry (electron ionization) analysis and infrared spectroscopy. They show enantiotropic nematic behavior in a broad temperature range, confirmed by a polarizing thermomicroscopy and differential scanning calorimetry. Detailed synthetic procedures are attached. Synthesized compounds show a significantly reduced absorption in the near-infrared (NIR) and medium-wavelength infrared (MWIR) radiation range, and stand as promising components of medium to highly birefringent liquid crystalline mixtures.

## 1. Introduction

Organic optical materials are used to construct many elements of optical devices operating in various spectral ranges, from ultraviolet [1,2,3,4,5] through visible [6,7] to infrared [8,9,10,11,12,13]. Most organic materials are passive elements; however, one group—mesomorphic materials—constitute the heart of active elements, where the wide tunable abilities of these materials are used. The near-infrared range (NIR), so important in fiber-optic communication, was not the main spectral area for which organic mesomorphic materials were developed. Instead, they were developed for the visible range and the entire display industry associated with it [14,15,16,17,18,19,20,21]. Therefore, all organic materials, especially mesomorphs, used in the near-infrared range, are not materials dedicated to this range. They show a number of disadvantages, such as parasitic absorption (the presence of absorption originating from overtones and combinational bands, primarily related to the vibration of C-H bonds in rings and alkyl chains) and indirectly related changes in optical signal depolarization [22,23,24]. Despite the fact that the absorption coefficients, related to higher than the first harmonic, have a lower level of magnitude, they still deteriorate the optical density of the liquid crystalline medium in NIR.

In order to reduce the absorption we decided to increase the weight of one of the most critical elements—hydrogen—by replacing the C-H bonds with C-D, which reduces the frequency of the fundamental vibration and shifts the absorption bands toward longer wavelengths [14,24,25,26,27,28]. The reduced mass causes such polyatomic systems, R-D or R-H, to be strongly affected, due to the high relative mass difference between protons and deuteriums. This in turn translates to the molecular vibration frequency shifts of fundamental frequencies (3000 cm^−1^) and higher harmonics (1700 nm) towards longer wavelengths, 2300 cm^−1^ and 2000 nm, respectively. This causes the optical density in important NIR window to be significantly lowered. Moreover, the deuteration of mesogenic molecules, as well as the fluorination, and/or chlorination approaches were proposed to shift the vibration bands beyond the spectrum of interest. The vibration bands of CF, CF_2_ and CF_3_ bonds occur at 7~9 μm. A modification of the fluorination solution is to propose the chlorination of liquid crystal molecules. This also allows for shifts of the vibration bands and overtones outside the area of interest [23,29,30,31]. Replacing fluorine with a heavier chlorine atom causes the C-Cl vibration wavelength to occur between approximately 12.5 and 15.4 μm [32]. While the intensity of the C-F overtones is relatively small, though still noticeable in the IR region, the overtone wavelength of C-Cl bonds is now longer than 6 μm, and therefore cleans up the window in the MWIR and LWIR regions. Nevertheless, the replacement of elastic aliphatic chains with short perfluorinated or chlorinated groups in liquid crystal molecules has a drastic effect on the presence of the mesophase. However, the liquid crystallinity decay problem for the fluorinated molecules is currently solved. There are many organic structures that exhibit temperature-wide nematic phases, even though they lack the flexibility and aspect ratio required previously.

The optical density of liquid crystal which is the active medium of many non-display optical devices is one of the key factors, either in high-power laser applications, [33,34] or in devices working at NIR and MWIR frequencies, especially those for which the working bands are close to C-H vibrations. High red shift observed for partially deuterated or perdeuterated materials can be exploited for enhancing the operating windows of “regular” nondeuterated liquid crystals. Dedicated materials for specific applications in the near-infrared range are the subject of our research. The possibility of extending the use of liquid crystals to NIR light could be very important in different areas, like optical fiber communications, with standard windows operating at 800–980 nm, 1300–1330 nm, and 1550–1670 nm, or in biomedical imaging, where the conventional therapeutical windows are centered at ranges of 650–950 nm (first window) or 1100–1350 nm (second window).

Liquid crystal devices can control the wavelength, polarization and phase, so they can be used to improve the performance of optical imaging systems even for biomedical applications, e.g., LC polarization controllers for bio-imaging, where LCs act as a simple variable retarder creating and controlling polarization changes [35,36,37,38]. LC-tunable filters were successfully demonstrated in research labs and some industrial environments to have a great potential in agriculture, process inspection, remote sensing and medical diagnosis for spectral and polarization imaging (SPI) and hyperspectral imaging (HSI). For those applications, the interest is usually in the near-infrared window (750–1300 nm), which is covered with LC devices, enhancing the functionality of optical imaging systems [39,40,41,42,43,44]. LCs can also be applied in optical systems based on a LCoS-SLM, that acts as a waveplate with a programmable spectra retardance function in the visible and NIR wavelength [45,46]. LCs can be applied to control the tuning of the metamaterial electromagnetic response in NIR in metamaterial–liquid crystal cell structures [20,47].

**Figure 1:**
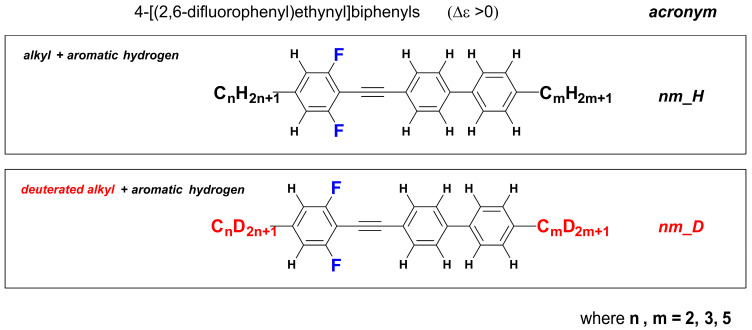
General structures of investigated materials.

In this work we focused our attention around strongly nematogenic tolanes, from 4-[(2,6-difluorophenyl)ethynyl]biphenyls family—see Figure 1. Lateral fluoro-substitution, in particular a 2,6-difluorophenyl acetylene unit, offers unique and valuable nematic stability [48,49,50,51,52] together with increased birefringence. Although the structures itself are already known [50,51] we present a totally new, synthetic approach to the material which reflects the idea of reducing IR absorption by increasing the mass of the components of the molecule.

## 2. Materials and Methods

### 2.1. Materials

The genes 1-Bromo-3,5-difluorobenzene and 4-bromo-biphenyl[1,1′] were purchased from Fluorochem (Hadfield, UK) and used as received. The genes 1,8-diazabicyclo(5.4.0)undec-7-ene, and 2-methylbut-3-yn-2-ol were purchased from Acros Organics, (Geel, Belgium) and used as received. Toluene, acetone, hydrochloric acid, sodium nitrite, potassium iodide, anhydrous potassium carbonate and copper(I) iodide were purchased from Avantor Performance Materials Poland S.A (Gliwice, Poland) and used as received. Palladium(II) acetate, bis(triphenylphosphine)palladium(II) chloride, and sodium hydride (60% dispersion in mineral oil) were purchased from Sigma-Aldrich Sp.z.o.o (Poznan, Poland) and used as received. THF was distilled from sodium ketyl radical under nitrogen atmosphere prior to use. Methyl iodide-d3 and heavy water D_2_O were purchased from Armar AG (Dottingen, Switzerland).

The main source of deuterium we decided to use in this work was the deuterium gas D_2_, in situ generated during the electrolysis of heavy water, D_2_O. The gas produced acted as a reactant in the palladium-catalyzed addition reactions to the carbon–carbon triple bond. A H-Cube Pro (ThalesNano, Budapest, Hungary) pressure flow reactor was used to carry out this process. Additionally, methyl iodide (d_3_) was used as the deuterium source, which formed a chain with an odd number of carbon atoms in nucleophilic substitution reactions.

### 2.2. Synthesis

**Figure 2:**
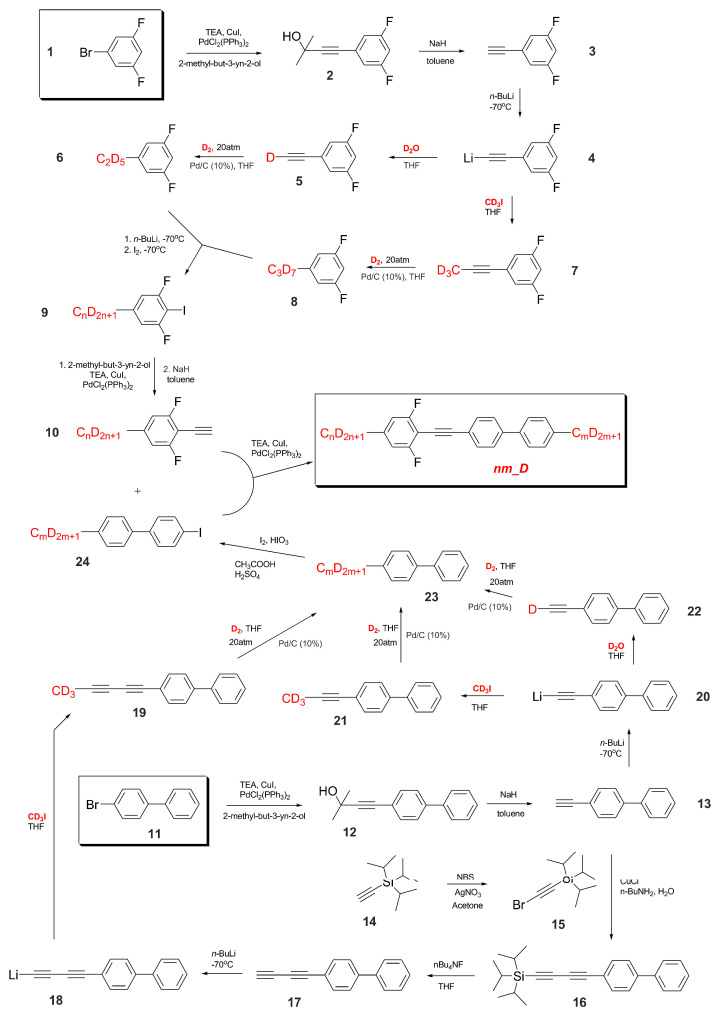
Synthesis of alkyl-deuterated tolanes ***nm_D***.

Synthetic process of alkyl-deuterated tolanes (with the acronym ***nm___D***) began with 1-bromo-3,5-difluorobenzene **1**, which was coupled to a triple carbon–carbon bond, resulting 1-ethynyl-3,5-difluorobenzene **3**—see Figure 2. Then the proper lithium acetylide **4** was created and reacted either with heavy water, D_2_O, or methyl iodide, *d*_3_—structure **5** and **7**, respectively. Both reacted with gaseous deuterium and formed the desired ethyl-*d*_5_ **6** and propyl-*d*_7_ **8** derivatives. Next ortho-directed metalation followed by iodination was performed **9**, and finally the Sonogashira coupling to a carbon–carbon triple bond, resulting in **10**. The right part of the tolane molecule was constructed using 4-bromobiphenyl **11** as a starting reagent and an analogous synthetic procedure. Additionally, we decided to create a pentyl-*d*_13_ homologue **23**, using the Cadiot–Chodkiewicz procedure for buta-1,3-diynyl derivative **17**.

Finally, the tolanes ***nm___D*** were created using the Sonogashira protocol with 2-ethynyl-1,3-difluoro-5-alkylbenzene **10** and 4-alkyl-4′-iodo-[1,1′]-biphenyl **24** as a substrates.

### 2.3. Mesomrphic Properties and Discussion

**Figure 3:**
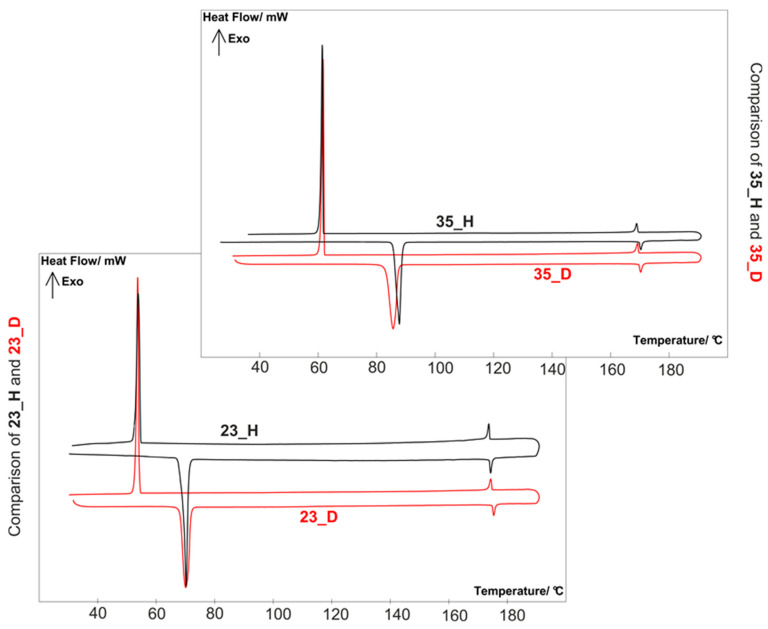
Comparison of DSC of selected hydrogen and deuterated structures.

The sequence of phase transitions and their temperatures were determined by polarizing optical microscopy with the ‘Olympus’ BX51 polarizing microscope (Shinjuku, Tokyo, Japan), equipped with a Linkam hot stage THMS-600 (Linkam Scientific Instruments Ltd., Tadworth, UK) and by differential scanning calorimetry using the DSC SETARAM 141 instrument (KEP Technologies Group’s DNA, Montauban, France) with the scanning rate 2 K min^−1^ in both heating and cooling cycles. The phase situation of alkyl-deuterated structures ***nm___D*** was correlated with their hydrogen isotopologues ***nm___H*_—_**Table 1 and Figure 3. Differential scanning calorimetry graphs of investigated compounds were gathered in Supplementary Materials.

Alkyl-deuterated structures showed similar nematic behavior compared to their hydrogen analogues; however, the temperature-wide nematic is seen for ***nm___D*** structures. This was due to the differences in crystalline–nematic transition temperatures, which were 1–3 °C lower, and nematic–isotropic liquid transitions temperatures, which were 1–3 °C higher for alkyl-deuterated structures. Correlation between enthalpies of melting was hard to extract, and there were no significant differences between investigated structures. Differential scanning calorimetry graphs of selected structures ***23___H***, ***23___D*** and ***35___H***, ***35___D*** are shown in Figure 3.

### 2.4. Optical Properties and Discussion

Refractive indices of neat materials were measured by the Metricon Model 2010/M Prism Coupler (Metricon Corporation, Pennington, NJ, USA), equipped with 443 nm, 636 nm and 1550 nm lasers. Samples of liquid crystals were placed on glass plates rubbed with SE-130 polyimide (Nissan Chemical^®^, Tokyo, Japan). Ordinary refractive index n_o_ and extraordinary refractive index n_e_ were measured separately using transverse-magnetic TM and transverse-electric TE polarization of incident beams. Samples were measured at elevated temperatures (5 Kelvin steps), with the limit of the setup at 200 °C. Exact values of refractive indices n_o_, n_e_ for three wavelengths 443 nmn, 636 nm, and 1550 nm were collected in the Supplementary Materials.

**Figure 4:**
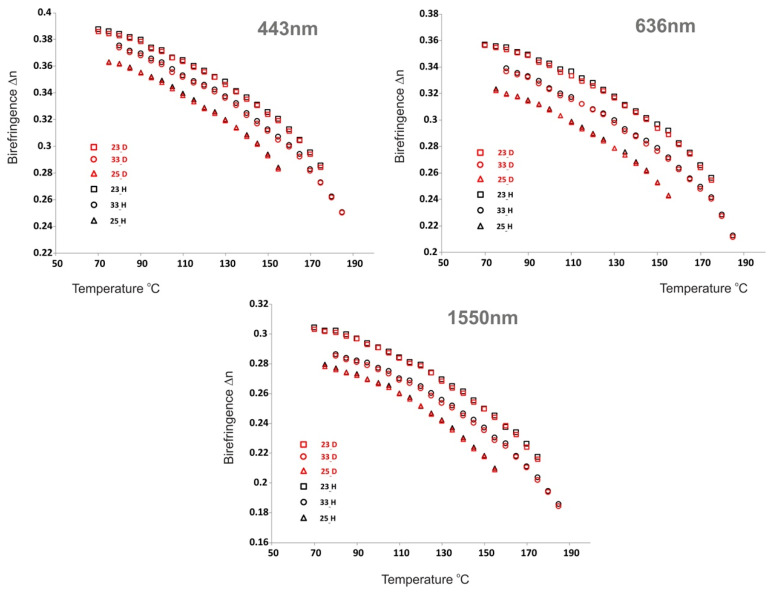
Temperature dependence of birefringence measured for ***23___H***, ***33___H***, ***25___H***, ***23___D***, ***33___D***, and ***25___D*** at λ = 443, 636, and 1550 nm.

Both, deuterated structures ***nm___D*** and their hydrogen isotopologues ***nm___H*** showed close properties, when comparing homologues with the same number of carbon atoms. The differences in the values of refractive indices were very small, 0.001 on average, while it should be noted that the hydrogen structures ***nm___H*** had slightly higher values of refractive indices n_o_ and n_e_. Therefore, minimum values differences were perceived in birefringence Δn (0.001 average)—See Figure 4.

#### 2.4.1. Medium Wavelength Infrared MWIR Absorption (IR-C)—2500–17,000 nm (4000–600 cm^−1^) Range

Measurements of infrared spectra using the transmittance FT-IR technique, with a carbon tetrachloride as solvent at a concentration of 0.1 M, were performed on the Nicolet iS10 Spectrometer Thermo Scientific (Thermo Fisher Scientific, Waltham, MA, USA). The spectra were measured in the range of 4000–1000 cm^−1^ with 32 scans and a resolution of 4 cm^−1^. The windows in cuvette for testing the solutions were made of CaF_2_ and the cuvette thickness was 0.5 mm. Before each sample measurement, a background measurement for pure solvent and air in the measuring chamber was recorded.

**Figure 5:**
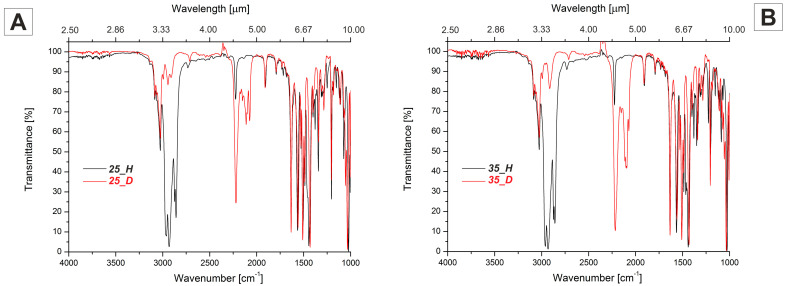
FT-IR spectra of selected isotopologues: (**A**) ***25_H*** and ***25_D***, and (**B**) ***35_H*** and ***35_D***.

Regardless of the number of carbon atoms in terminal chain, FT-IR spectra of hydrogen compounds were very similar (see Figure 5). Bands at about 2970 cm^−1^, 2870 cm^−1^ and 2930 cm^−1^, 2850 cm^−1^ could be assigned to the asymmetric and symmetric stretching vibration between carbon and hydrogen atom in group CH_3_ and CH_2_, respectively. Bands just above 3000 cm^−1^ were associated with stretching vibrations between carbon and hydrogen atoms in benzene rings. Bands at about 2210 cm^−1^ were connected with the stretching vibration of triple bonds between carbon atoms. Bands at about 1900 cm^−1^ could be assigned to the vibration of benzene rings. Bending vibration of functional groups inside studied compounds were visible as bands below 1700 cm^−1^. As expected, the biggest difference in FT-IR spectra of deuterated isotopologues was the shift of bands from stretching C-H vibrations in aliphatic chains, toward lower wavenumber values (between 2220–2050 cm^−1^). Due to the non-100% deuterium introduction efficiency in the aliphatic chains, a small band at around 2930 cm^−1^ was also observed. Nevertheless, the transmittance in the region between 3000–2500 cm^−1^ was above 80% for the compound ***25_D,*** as well as ***35_D***, and was found to be much higher than in its hydrogen counterparts, ***25_H*** and ***35_H***. It showed the potential advantage of studied deuterated compounds in near-infrared applications where overtones of stretching vibrations in aliphatic chains were undesirable.

#### 2.4.2. Near-Infrared NIR Absorption (IR-A + IR-B)—800–2000 nm (12,500–5000 cm^−1^) Range

The study of spectral characteristics in the near-infrared range (800–2000 nm) was carried out using the Shimadzu UV-Vis-NIR 3600 spectrophotometer (Shimadzu, Kyoto, Japan) using the transmission technique, in solutions. In transmission techniques, the oscillation spectrum was received by measuring the intensity of the radiation after it passed through the sample, where a decrease in the intensity of the incident beam indicated the absorption of the radiation. The spectrophotometer was equipped with three detectors, two of them were used to conduct measurements in the near-infrared range: indium–germanium–arsenic photodiode (InGaAs) and a cooled lead sulphide detector. The solutions of the tested compounds were prepared at concentrations of 0.5 M. Carbon tetrachloride was used as a solvent and spectra were recorded in quartz cuvettes with a volume of 1 mL with an optical path equal to 1 cm, made of black glass eliminating the phenomenon of internal reflection.

**Figure 6:**
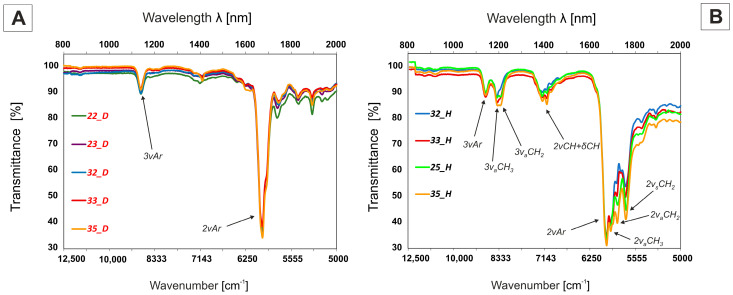
NIR spectra of deuterated compounds ***nm___D*** (**A**) and their hydrogen isotopologues ***nm___H*** (**B**) (c = 0.5 M), with *ν*-stretching vibrations, *ν_a_*-asymmetric stretching, *v_s_*-symmetric stretching, and *δ*-bending vibrations.

The overtones of C-H bond vibrations occured at a frequency approximately two or three times higher than the fundamental vibration and were approximately forty to two hundred times weaker. They had similar patterns to those in the MWIR, although there were some differences, primarily a wider separation with each overtone, caused by minimalization of symmetric vibrations, leaving the asymmetric bands more isolated. The consolidated spectrum of alkyl-deuterated tolanes ***nm___D*** clearly showed the major peak at 1670 nm, which was assigned to the first overtone of the aromatic C-H stretch and was a doublet representing a combination of four individual fundamental bands, only one of which was IR active. The second overtone of C-H stretching vibrations was measured at 1135 nm—Figure 6. The non-deuterated isotopologues, ***nm___H,*** had additional absorption bands derived from C-H stretching vibrations in the aliphatic chain. The first overtones of C-H stretching occurred between 1680 and 1800 nm and the second overtones between 1150 and 1220 nm, where absorption bands of the methyl, methylene and methine groups occurred at different wavelengths. The first overtone of CH_3_ asymmetrical and symmetrical stretches occurred at 1692 and 1700 nm, respectively. The asymmetric and symmetric CH_2_ stretching bands appeared at approximately 1720 and 1760 nm, respectively. In the area of the second overtone, absorption was observed at 1185 nm and 1200 nm, corresponding to the vibrations in the CH_3_ and CH_2_ groups. The region of 1350–1430 nm was assigned as C-H combination bands: 2 × CH stretch and CH bending of both CH_2_ and CH_3_ groups in the aliphatic chain.

## 3. Conclusions

In this work we showed the synthetic methodology, mesomorphic behavior and IR properties of deuterated versions of known nematics from the phenyltolane family. In the synthesis process, the main emphasis was placed on the use of the continuous flow reactor, where deuterated parts of molecules were formed. Isotopic pure-alkyl parts of molecules were created using the D-hydrogenolysis procedure of D_2_ gas to CC triple bonds. The synthesis approach was successfully designed to minimize the number of deuterium sources and to rely as much as possible or solely on heavy water, which was the cheapest and most accessible deuterium source. Deuterated versions of liquid crystals showed almost identical properties to their hydrogen analogues both from the mesomorphic and optical (refractive indices) point of view. Of course, there were slight differences in the temperatures of phase transitions and the values of refractive indices, but these structures could still be twin-classified—at least from the mesomorphic and optical point of view. This should be considered as an advantage, since it was possible to predict the properties of deuterated structures without the need to synthetically obtain them. The cost of obtaining deuterated structures was much higher compared to the production of their hydrogen equivalents. The differences in properties that were clearly visible for this type of material could only be detected in the absorption of IR radiation. The deuterated versions of the liquid crystals that were investigated in this work showed significantly reduced absorption especially in the NIR range. This was the advantage of deuterated materials over hydrogen equivalents, especially when it came to the specific applications of liquid crystals beyond the visible spectrum.

## Figures and Tables

**Table 1:** Phase transition temperatures (°C) and enthalpies (kJ·mol^−1^) from DSC measurements obtained during heating cycles.

Acronym	Phase Transition Temperatures [°C] (*Enthalpy* [kJ mol^−1^])	Temperature Range of Nematic Phase [°C]
***nm___D***	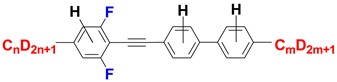	
***22_D***	Cr **107.2** *(23.0)* N **163.9** Iso	56.7
***23_D***	Cr **68.1** *(17.5)* N **174.8** Iso	106.7
***32_D***	Cr **73.2** *(24.7)* N **179.3** Iso	106.1
***33_D***	Cr **78.0** *(26.6)* N **186.8** Iso	108.8
***25_D***	Cr **72.0** *(20.0)* N **160.3** Iso	88.3
***35_D***	Cr **85.6** *(24.8)* N **170.3** Iso	84.7
***nm___H***	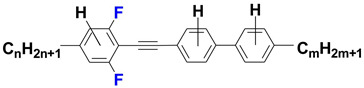	
***22_H***	Cr **108.9** *(22.7)* N **165.4** Iso	56.5
***23_H***	Cr **70.2** *(18.8)* N **173.8** Iso	103.6
***32_H***	Cr **74.7** *(26.9)* N **175.2** Iso	100.5
***33_H***	Cr **78.3** *(26.9)* N **186.5** Iso	108.2
***25_H***	Cr **73.6** *(17.6)* N **159.6** Iso	86.0
***35_H***	Cr **87.8** *(24.1)* N **169.7** Iso	81.9

## Data Availability

Data are contained within the article or supplementary materials.

## Supplementary Materials

The following are available online at https://www.mdpi.com/article/10.3390/ma14164653/s1, Figure S1: DSC spectrum of compound ***22_D***, Figure S2: DSC spectrum of compound ***22_H***, Figure S3: DSC spectrum of compound ***23_D***, Figure S4: DSC spectrum of compound ***23_H***, Figure S5: DSC spectrum of compound ***25_D***, Figure S6: DSC spectrum of compound ***25_H***, Figure S7: DSC spectrum of compound ***32_D***, Figure S8: DSC spectrum of compound ***32_H***, Figure S9: DSC spectrum of compound ***33_D***, Figure S10: DSC spectrum of compound ***33_H***, Figure S11: DSC spectrum of compound ***35_D***, Figure S12: DSC spectrum of compound ***35_H***, Figure S13. MS spectrum of compounds ***22_H*** and ***22_D***, Figure S14. MS spectrum of compounds ***23_H*** and ***23_D***, Figure S15. MS spectrum of compounds ***25_H*** and ***25_D***, Figure S16. MS spectrum of compounds ***32_H*** and ***32_D***, Figure S17. MS spectrum of compounds ***33_H*** and ***33_D***, Figure S18. MS spectrum of compounds ***35_H*** and ***35_D***, Figure S19. IR spectrum of compound ***22_H***, Figure S20. IR spectrum of compound ***22_D***, Figure S21. IR spectrum of compound ***23_H***, Figure S22. IR spectrum of compound ***23_D***, Figure S23. IR spectrum of compound ***25_H***, Figure S24. IR spectrum of compound ***25_D***, Figure S25. IR spectrum of compound ***32_H***, Figure S26. IR spectrum of compound ***32_D***, Figure S27. IR spectrum of compound ***35_H***, Figure S28. IR spectrum of compound ***35_D***, Figure S29. ^1^H NMR spectrum of compound ***22_D***, Figure S30. ^1^H NMR spectrum of compound ***23_D***, Figure S31. ^1^H NMR spectrum of compound ***32_D***, Figure S32. ^1^H NMR spectrum of compound ***33_D***, Figure S33. ^1^H NMR spectrum of compound ***35_D***, Figure S34. POM texture of nematic phase of compound ***33_D***, Figure S35. POM texture of nematic phase of compound ***33_D***, Figure S36. POM texture of nematic phase of compound ***25_D***, Figure S37. POM texture of nematic phase of compound ***35_D***, Table S1. Measured refractive indices (n_e_ and n_o_) of ***33_D*** at λ = 443, 636, and 1550 nm, and at different temperatures, Table S2. Measured refractive indices (n_e_ and n_o_) of ***33_H*** at λ = 443, 636, and 1550 nm, and at different temperatures, Table S3. Measured refractive indices (n_e_ and n_o_) of ***23_D*** at λ = 443, 636, and 1550 nm, and at different temperatures, Table S4. Measured refractive indices (n_e_ and n_o_) of ***23_H*** at λ=443, 636, and 1550 nm, and at different temperatures, Table S5. Measured refractive indices (n_e_ and n_o_) of ***25_D*** at λ = 443, 636, and 1550 nm, and at different temperatures, Table S6. Measured refractive indices (n_e_ and n_o_) of ***25_H*** at λ = 443, 636, and 1550 nm, and at different temperatures.
