# Supplementary material for: Synthesis, Mesomorphism and the Optical Properties of Alkyl-deuterated Nematogenic 4-[(2,6-Difluorophenyl)ethynyl]biphenyls
Source: Materials (Basel). 2021 Aug 18;14(16):4653. doi: 10.3390/ma14164653 (PMC8399011; doi:10.3390/ma14164653)
Supplement: Supplementary file 1 [file materials-14-04653-s001.zip › materials-1313719-supplementary.pdf]

materials MDPI

Supplementary

Synthesis, Mesomorphism and the Optical Properties of Alkyl-deuterated Nematogenic 4-[(2,6-difluorophenyl)ethynyl]biphenyls

Jakub Herman, Piotr Harmata, Michał Czerwiński, Olga Strzeżysz, Marta Pytlarczyk, Monika Zając and Przemysław Kula *

Faculty of Advanced Technologies and Chemistry, Military University of Technology, 2 Gen. S. Kaliskiego St., 00-908 Warsaw, Poland; jakub.herman@wat.edu.pl (J.H.); piotr.harmata@wat.edu.pl (P.H.); michal.czerwinski@wat.edu.pl (M.C.); olga.strzezysz@wat.edu.pl (O.S.); marta.pytlarczyk@wat.edu.pl (M.P.); monika.zajac@wat.edu.pl (M.Z.)

* Correspondence: przemyslaw.kula@wat.edu.pl

Flow D-Hydrogenations in the Continuous Flow H-Cube Pro reactor.

General Procedure: A solution was prepared in a conical flask with an appropriate starting material with a concentration of 0.05–0.1 M in a suitable solvent (Methanol (MeOH), Tetrahydrofuran (THF), MeOH + THF). The reaction parameters (temperature, flow rate and deuterium pressure) were selected on the H-Cube controller. A reactor equipped with a suitable CatCart loading (10 wt% Pd/C: 30 mm or 70 mm length) was initially run on a pure solvent pump system until the instrument reached the desired reaction parameters and stable processing was ensured. After the system was stabilized the sample inlet line was switched to the vial containing the substrate. The reaction was monitored by constant GC analysis of the processed reaction mixture. After the reaction was finished the system was again run on a pure solvent pump for an additional 10 min until the cartridge system was washed (in order to remove any substrate or product or impurity adsorbed on the catalyst loading). Deuterated products were obtained after the evaporation of solvents.

1-ethynyl-3,5-difluorobenzene 3

1-bromo-3,5-difluorobenzene **1** (193 g; 1.0 mol), triethylamine TEA (139 mL ; 1.0 mol), 1,8-Diazabicyclo[5.4.0]undec-7-ene DBU (150 mL; 1.0 mol), Bis(triphenylphosphine)palladium(II) dichloride or PdCl2(PPh3)2 (0.3 mol%), Copper (I) iodide or CuI (0.15 mol%) and toluene (700 mL) were mixed and heated to refluxed. Then 2-methylbut-3-yn-2-ol (93.0 g; 1.1 mol) was added dropwise and the reaction mixture was stirred at reflux for 1 h. After the reaction was completed, the mixture was washed with water x3, dried over $MgSO_4$ and solvent evaporated. Crude product **2** was distilled at reduced pressure. Boiling point B.p. = 75–77 °C (0.4 mmHg)- yield 180 g (85%). Protected acetylene **2** from the previous step and anhydrous toluene (500 mL) were mixed and catalytic amount of sodium hydride (6% mol) was added. The mixture was stirred under reflux while acetone was distilled off. When the reaction was completed, crude product was purified using column chromatography (silica gel and toluene as an eluent). Toluene was evaporated and crude product was distilled at reduced pressure. Boiling point B.p. = 68 °C (60 mmHg), yield 83.0 g (60%), Mass Spectrum (Electron Ionisation) or MS (EI) m/z: 138 (M+); 118; 112.

1-ethynyl-3,5-difluorobenzene-d_1 5

1-ethynyl-3,5-difluorobenzene **3** (25 g; 0.18 mol) and anhydrous tetrahydrofuran (200 mL) were mixed and cooled to −70 °C. Then the *n*-buthyllithium (0.18 mol) solution in hexane was added dropwise to the reaction at −70 °C. The reaction mixture was stirred in

this temperature for 2 h. Next the mixture of D_2O (4 g; 0.18 mol) and anhydrous tetrahydrofuran (10 mL) was added dropwise and the reaction was warmed to room temperature. Product **1-ethynyl-3,5-difluorobenzene-*d*$_1$ 5** was purified using column chromatography (silica gel and THF) and as a solution further used. MS (EI) m/z: 139 (M+); 113; 94.

1-ethyl-3,5-difluorobenzene-*d*$_5$ 6

Flow D-Hydrogenations in the Continous Flow (CF) H-Cube Pro reactor. Conditions: concentration C = 0.05 M in THF, temperature T = 40 °C, D_2 pressure p = 20 bar, Pd/C (10 wt%) cartridge system length 30 mm, flow rate 1 mL/min. Product **6** was purified using distillation under atmospheric pressure. Boiling point B.p. = 137 °C (760 mmHg), yield 20.7 g (78%), MS(EI) m/z: 147 (M+); 129; 115; 102.

1,3-difluoro-5-(prop-1-ynyl)benzene-*d*$_3$ 7

1-ethynyl-3,5-difluorobenzene **3** (40 g; 0.29 mol) and anhydrous tetrahydrofuran (300 mL) were mixed and cooled to −70 °C. Then the *n*-buthyllithium (0.29 mol) solution in hexane was added dropwise to the reaction at −70 °C. The reaction mixture was stirred in this temperature for 2 h. Next the mixture was warmed to room temperature and a solution of methyl iodide-*d*$_3$ (42 g; 0.29 mol) and anhydrous tetrahydrofuran (80 mL) was added dropwise at this temperature. After addition the reaction was stirred for 1 h at room temperature. Then tetrahydrofuran was evaporated and crude product was treated with water and extracted with hexane. Organic layer was dried over $MgSO_4$ and product **7** was purified using distillation under reduced pressure. Boiling point B.p. = 80–82 °C (15 mmHg), yield 34.0 g (76%). MS (EI) m/z: 155 (M+); 136; 126.

1,3-difluoro-5-propylbenzene-d$_7$ 8

Flow D-Hydrogenations in the CF H-Cube Pro reactor. Conditions: concentration C = 0.05 M in MeOH, temperature T = 40 °C, D_2 pressure p = 40 bar, Pd/C (10 wt%) cartridge system length 30 mm, flow rate 1 mL/min. Product **8** was purified using distillation under atmospheric pressure. Boiling point B.p. = 140–142 °C (760 mmHg), yield 24 g (67%), MS (EI) m/z: 163 (M+); 145; 129; 109; 102.

5-ethyl-1,3-difluoro-2-iodobenzene-*d*$_5$ 9a

1-ethyl-3,5-difluorobenzene-*d*$_5$ **6** (20.7 g; 0.14 mol) was mixed with anhydrous THF (250 mL) under nitrogen and cooled to −78 °C in an acetone/dry ice bath. Solution of *n*-buthyllithium dissolved in cyclohexane–hexane mixture (0.155 mol ; C = 2.5 M) was added dropwise and temperature was kept below −70 °C. The reaction mixture was stirred for 2.5 h in −78 °C. Then a solution of iodine (40 g; 0.155 mol) in anhydrous THF was added dropwise and temperature was kept below −70 °C. The reaction mixture was allowed to reach room temperature. Saturated solution of Na_2SO_3 was added to the mixture to get rid of iodine excess. Then tetrahydrofuran was evaporated and crude product was treated with water and extracted with hexane. Organic layer was dried over $MgSO_4$ and concentrated on a rotary evaporator. Crude product was distilled at reduced pressure. Boilin point B.p. = 115–120 °C (15 mmHg), yield 33.3 g (85%). MS (EI) m/z: 273 (M+); 255; 241; 128.

1,3-difluoro-2-iodo-5-propylbenzene-d$_7$ 9b

Boiling point B.p. = 78–80 °C (1 mmHg), yield 24.1 g (83.3%). MS (EI) m/z: 289 (M+); 255; 241; 128.

5-ethyl-2-ethynyl-1,3-difluorobenzene-d5 10a

Compound synthesized using analogous procedure as described for **3**. Boiling point B.p. = 80–82 °C (10 mmHg), yield 15.6 g (74.0%). MS (EI) m/z: 171 (M+); 153; 139; 126.

2-ethynyl-1,3-difluoro-5-propylbenzene-*d*$_7$ 10b

Boiling point B.p. = 82–83 °C (5 mmHg), yield 7.5 g (77.7%). MS (EI) m/z: 187 (M+); 153; 139; 126.

4-ethynylbiphenyl 13

Compound synthesized using analogous procedure as described for **3**. Melting temperature M.p. = 86–87 °C, yield 56.0 g (63.0%). MS (EI) m/z: 178 (M+); 152; 139; 126.

(bromoethynyl)(triisopropyl)silane 15

Ethynyl(triisopropyl)silane **14** (22 g; 0.12 mol), N-Bromosuccinimide NBS (23 g; 0.126 mol), silver (I) nitrate (2 g; 0.012 mol) and anhydrous tetrahydrofuran (200 mL) were stirred at room temperature for 2 h. Mixture was washed with water x3, product extracted with dichloromethane. After the reaction was completed dichloromethane was added, organic layer was washed with water ×3, separated, dried over $MgSO_4$ and concentrated. Crude product as an yellow liquid was later used without further purification. Yield 27.0 g (87.0%). MS (EI) m/z: 262, 260 (M+); 219, 217; 191, 189; 163, 161; 149, 147.

(4-biphenyl-4-ylbuta-1,3-diyn-1-yl)(triisopropyl)silane 16

Copper(I) chloride (0.3 g; 0.003 mol) and solution of *n*-buthylamine (44 g; 0.6 mol) in water (140 mL) were mixed at room temperature. After 0.5 h hydroxylamine hydrochloride (0.7 g; 0.01 mol) was added at room temperature and the reaction was cooled to 0 °C. At this temperature 4-ethynylbiphenyl **13** (17.8 g; 0.1 mol) was added in portions. Reaction was stirred for 3 h at 0 °C. Next a solution of (4-biphenyl-4-ylbuta-1,3-diyn-1-yl)(triisopropyl)silane **16** (27 g; 0.105 mol) in THF (30 mL) was added dropwise at 0 °C. After the addition reaction was stirred for 2 h at 0 °C and then at room temperature for 5 h. A solution of NH_4Cl was added to reaction and product was extracted with toluene, organic layer was washed with water ×3, dried over $MgSO_4$ and concentrated on a rotary evaporator. Product was recrystallized form MeOH. Slight yellow crystals, melting temperature M.p. = 86–87 °C, yield 28.6 g (80.0%). MS (EI) m/z: 358 (M+); 315; 287; 273; 259; 245; 229; 205; 181; 121.

4-buta-1,3-diyn-1-ylbiphenyl 17

Flask containing (4-biphenyl-4-ylbuta-1,3-diyn-1-yl)(triisopropyl)silane **16** (28.5 g; 0.08 mol) and anhydrous tetrahydrofuran (200 mL) was stirred at 0 °C. A solution of tetra-n-butylammonium fluoride TBAF (24.5 g; 0.087 mol) in anhydrous THF (100 mL) was added dropwise at 0 °C. After 2 h a solution of NH_4Cl was added to reaction and product was extracted with toluene, organic layer was washed with water ×3, dried over $MgSO_4$ and concentrated on a rotary evaporator. Product was recrystallized form MeOH. Brown crystals, melting temperature M.p. = 103–105 °C, yield 12 g (74.0%). MS (EI) m/z: 202 (M+); 174; 150; 101.

4-penta-1,3-diyn-1-ylbiphenyl-*d*$_3$ 19

4-buta-1,3-diyn-1-ylbiphenyl **17** (12.0 g; 0.06 mol) was mixed with anhydrous THF (150 mL) under nitrogen and cooled to −78 °C in an acetone/dry ice bath. Solution of *n*-buthyllithium dissolved in cyclohexane–hexane mixture (0.066 mol; C = 2.5 M) was added dropwise and temperature was kept below −70 °C. The reaction mixture was stirred for 2.5 h in −78 °C. Next the mixture was warmed to room temperature and a solution of methyl iodide-*d*$_3$ (9.3 g; 0.066 mol) and anhydrous tetrahydrofuran (10 mL) was added dropwise at this temperature. After addition the reaction was stirred for 1 h at room temperature. Then tetrahydrofuran was evaporated and crude product was treated with water and extracted with toluene. Organic layer was washed with water ×3, dried over $MgSO_4$ and concentrated on a rotary evaporator. Product was recrystallized form EtOH.

Brown crystals, melting temperature M.p. = 94–96 °C, yield 12 g (90.0%). MS (EI) m/z: 219 (M+); 190; 164; 142.

4-prop-1-yn-1-ylbiphenyl-*d_3* **21**

Compound synthesized using analogous procedure as described for **19**. Starting reagent was 4-ethynylbiphenyl **13** (30 g; 0.17 mol). Brown crystals, melting temperature M.p. = 68–69 °C, yield 30.0 g (90.0%). MS (EI) m/z: 195 (M+); 167; 152; 118.

4-ethynylbiphenyl-*d_1* **22**

Compound synthesized using analogous procedure as described for **5**. Crude 4-ethynylbiphenyl-*d_1* **22** was purified using column chromatography (silica gel and THF) and as a solution further used. MS (EI) m/z: 179 (M+); 152; 89.

4-ethylbiphenyl-*d_5* **23a**

Flow D-Hydrogenations in the CF H-Cube Pro reactor. Conditions: concentration C = 0.05 M in THF, temperature T = 40 °C, D_2 pressure p = 20 bar, Pd/C (10 wt%) cartridge system length 30 mm, flow rate 1 mL/min. Product **23a** was purified using distillation under reduced pressure. Boiling point B.p. = 98–101 °C (0.2 mmHg), yield 20.0 g (76%), MS (EI) m/z: 187 (M+); 169; 153; 116.

4-propylbiphenyl-*d_7* **23b**

Flow D-Hydrogenations in the CF H-Cube Pro reactor. Conditions: concentration C = 0.05 M in MeOH, temperature T = 40 °C, D_2 pressure p = 40 bar, Pd/C (10 wt%) cartridge system length 30 mm, flow rate 1 mL/min. Product **23b** was purified using distillation under reduced pressure. Boiling point B.p. = 118–120 °C (0.2 mmHg), yield 26 g (90%), MS(EI) m/z: 203 (M+); 169; 153; 129; 116; 102.

4-pentylbiphenyl-*d_{11}* **23c**

Flow D-Hydrogenations in the CF H-Cube Pro reactor. Conditions: concentration C = 0.05 M in MeOH, temperature T = 40 °C, D_2 pressure p = 50 bar, Pd/C (10 wt%) cartridge system length 70 mm, flow rate 1 mL/min. Product **23c** was purified using column chromatography (silica gel, hexane) and distillation under reduced pressure. Boiling point B.p. = 142–144 °C (0.3 mmHg), yield 14 g (90%), MS (EI) m/z: 235 (M+); 169; 154; 130; 116; 102.

4-ethyl-4'-iodobiphenyl-*d_5* **24a**

The mixture consisted of 4-ethylbiphenyl-*d_5* **23a** (19.0 g; 0.1 mol), I_2 (10.1 g ; 0.04 mol), HIO_3 (3.5 g; 0.02 mmol), glacial acetic acid (350 mL), water (80 mL) and concentrated sulphuric acid (10 mL) was stirred for 3 h at 80 °C. The mixture was poured into Na_2SO_3 water solution. The product was extracted to toluene, washed with water, dried over $MgSO_4$. Solvent was evaporated and the product was recrystallized form ethyl alcohol. Yellow crystals, melting temperature M.p. = 153–155 °C, yield 18.8 g (60%); MS (EI): 313 (M+), 295, 167, 152.

4-iodo-4'-propylbiphenyl-*d_7* **24b**

Melting temperature M.p. = 128–129 °C, yield 22.2 g (55%); MS (EI): 329 (M+), 295, 167, 152.

4-iodo-4'-pentylbiphenyl-*d_{11}* **24c**

Melting temperature M.p. = 111–113 °C, yield 11.2 g (53%); MS (EI): 361 (M+), 295, 167, 154.

4-ethyl-4'-[(4-ethyl-2,6-difluorophenyl)ethynyl]biphenyl-*d_{10}* *22_D*

Compound synthesized using analogous Sonogashira cross-coupling procedure as described for **2** and **3**. Starting reagents was 4-ethyl-4'-iodobiphenyl-d_5 **24a** (3 g; 0.01 mol), triethylamine TEA (1.3 mL ; 0.01 mol), 1,8-Diazabicyclo[5.4.0]undec-7-ene DBU (1.2 mL; 0.01 mol), PdCl2(PPh3)2 (0.3 mol%), CuI (0.15 mol%) and toluene (200 mL) as a solvent. 5-ethyl-2-ethynyl-1,3-difluorobenzene-d_5 **10a** (1.8 g; 0.011 mol) was added dropwise and the reaction mixture was stirred at reflux for 1 h. After the reaction was completed, the mixture was washed with water ×3, organic layer dried over $MgSO_4$ and solvent evaporated. Product was purified using the combination of column chromatography (silica gel and hexane) and recrystallization (ethyl alcohol) techniques. White crystals, melting temperature M.p. = 107.2 °C, yield 1.5 g (46.0%). Degree of deuteration: 98%. MS (EI) m/z: 356 (M+); 338; 320; 303; 178; 160.

4-[(4-ethyl-2,6-difluorophenyl)ethynyl]-4'-propylbiphenyl-d_{12} *23_D*

White crystals, melting temperature M.p. = 68.1 °C, yield 2.0 g (59.0%). Degree of deuteration: 97%. MS (EI) m/z: 372 (M+); 338; 320; 303; 186; 160.

4-[(2,6-. difluoro-4-propylphenyl)ethynyl]-4'-ethylbiphenyl-d_{12} *32_D*

White crystals, melting temperature M.p. = 73.2 °C, yield 1.7 g (53.0%). Degree of deuteration: 97%. MS (EI) m/z: 372 (M+); 354; 338; 320; 298; 186; 160.

4-[(2,6-difluoro-4-ethylphenyl)ethynyl]-4'-propylbiphenyl-d_{14} *33_D*

White crystals, melting temperature M.p. = 78.0 °C, yield 2.7 g (76.0%). Degree of deuteration: 97%. MS (EI) m/z: 388 (M+); 354; 320; 194; 160.

4-[(2,6-difluoro-4-pentylphenyl)ethynyl]-4'-ethylbiphenyl-d_{16} *25_D*

White crystals, melting temperature M.p. = 72.0 °C, yield 1.1 g (46.0%). Degree of deuteration: 95%. MS (EI) m/z: 404 (M+); 338; 320; 160.

4-[(2,6-difluoro-4-pentylphenyl)ethynyl]-4'-propylbiphenyl-d_{18} *35_D*

White crystals, melting temperature M.p. = 85.6 °C, yield 2.0 g (57.0%). Degree of deuteration: 95%. MS (EI) m/z: 420 (M+); 354; 320; 281; 207; 160.

Differential Scanning Calorimetry (DSC) Data

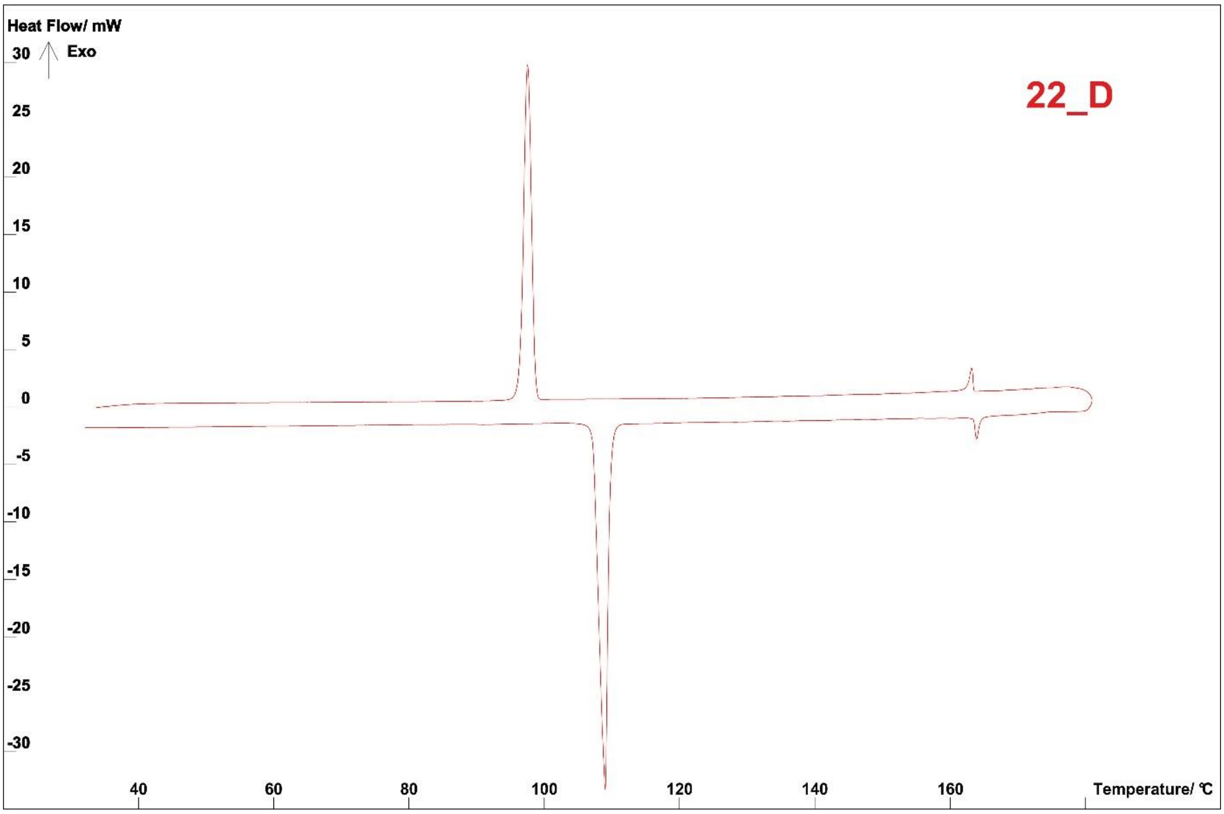

Figure S1. DSC spectrum of compound *22_D*.

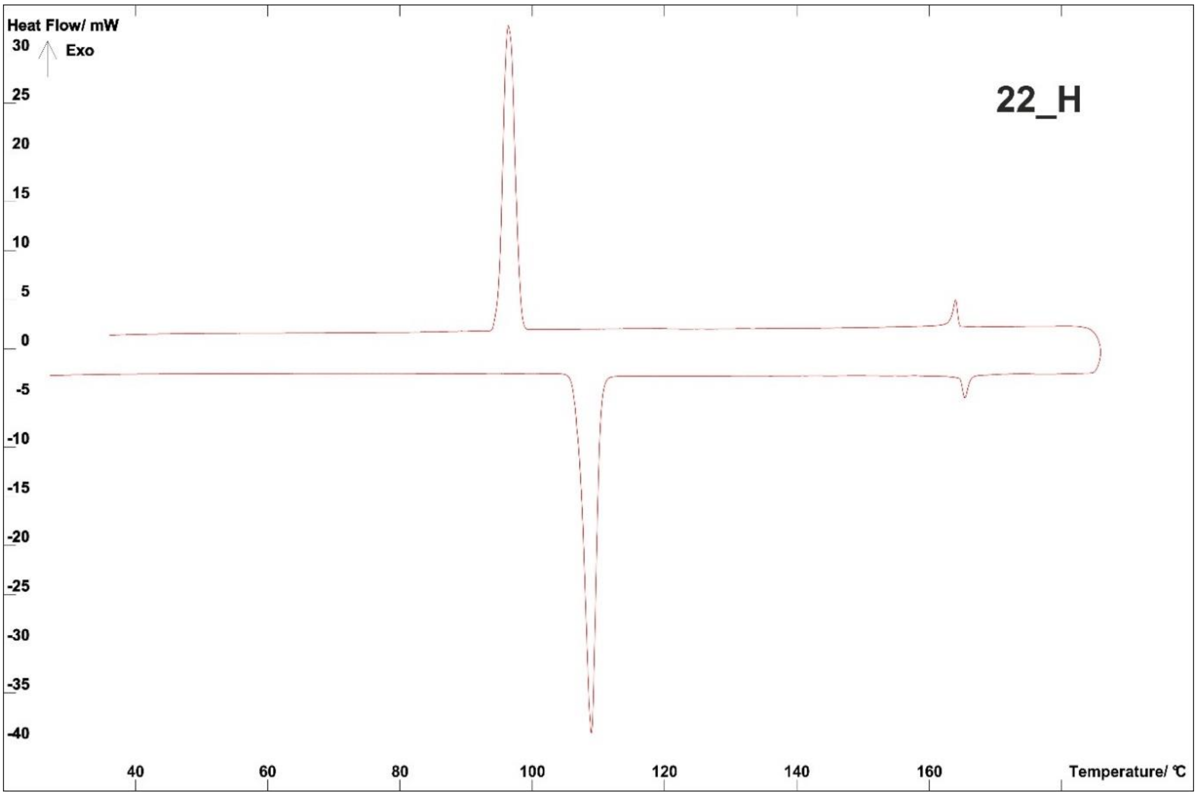

Figure S2. DSC spectrum of compound **22_H**.

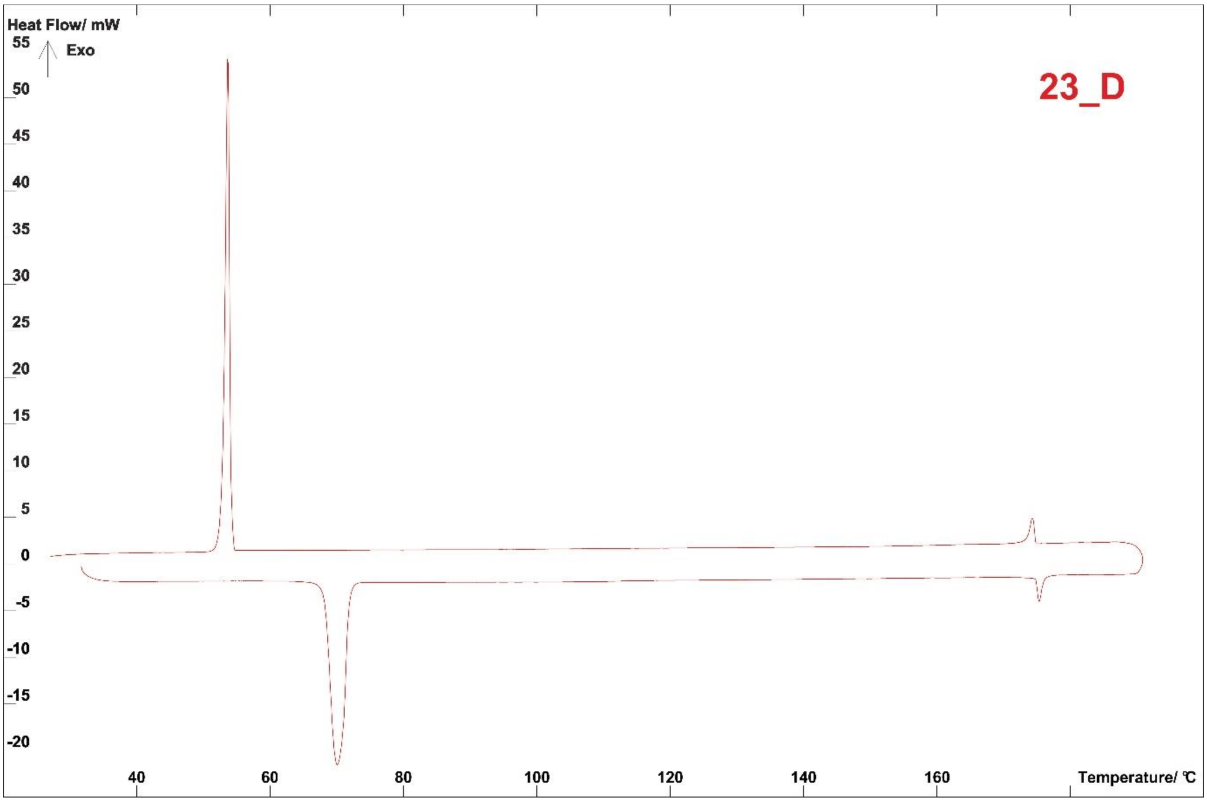

Figure S3. DSC spectrum of compound *23_D*.

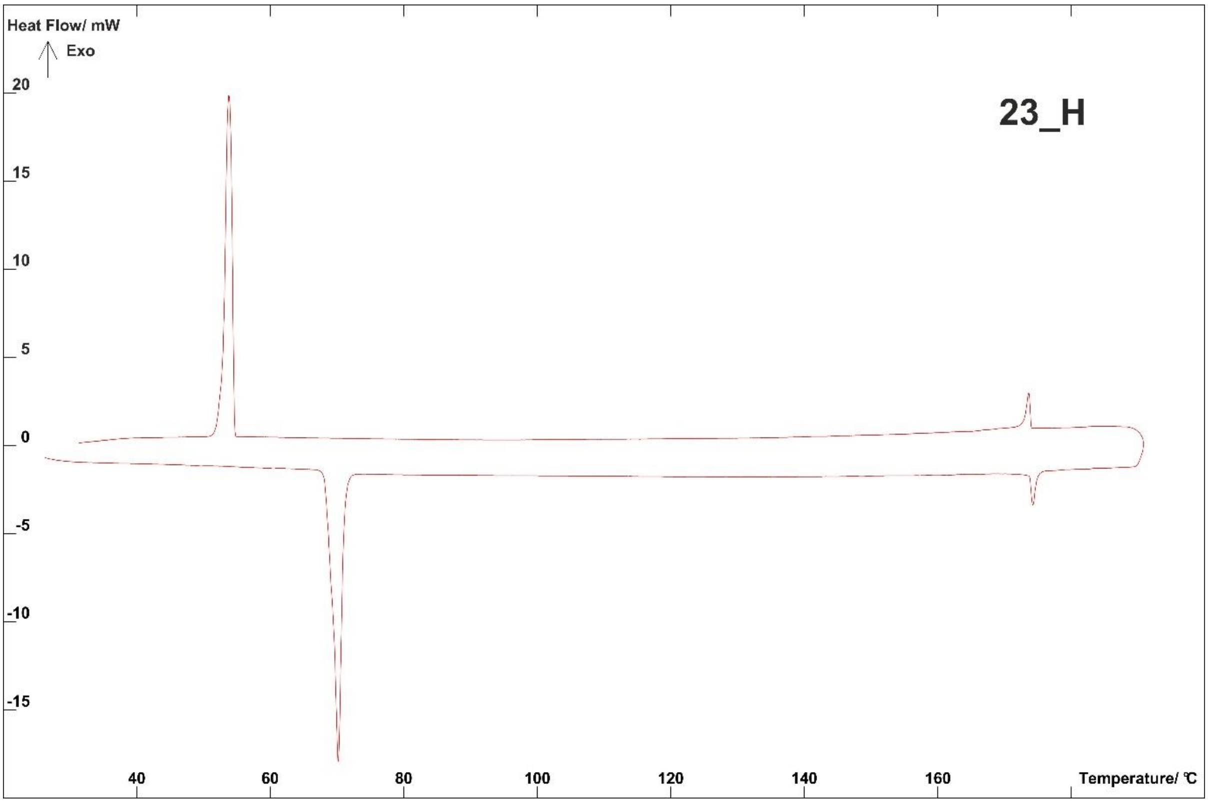

Figure S4. DSC spectrum of compound **23_H**.

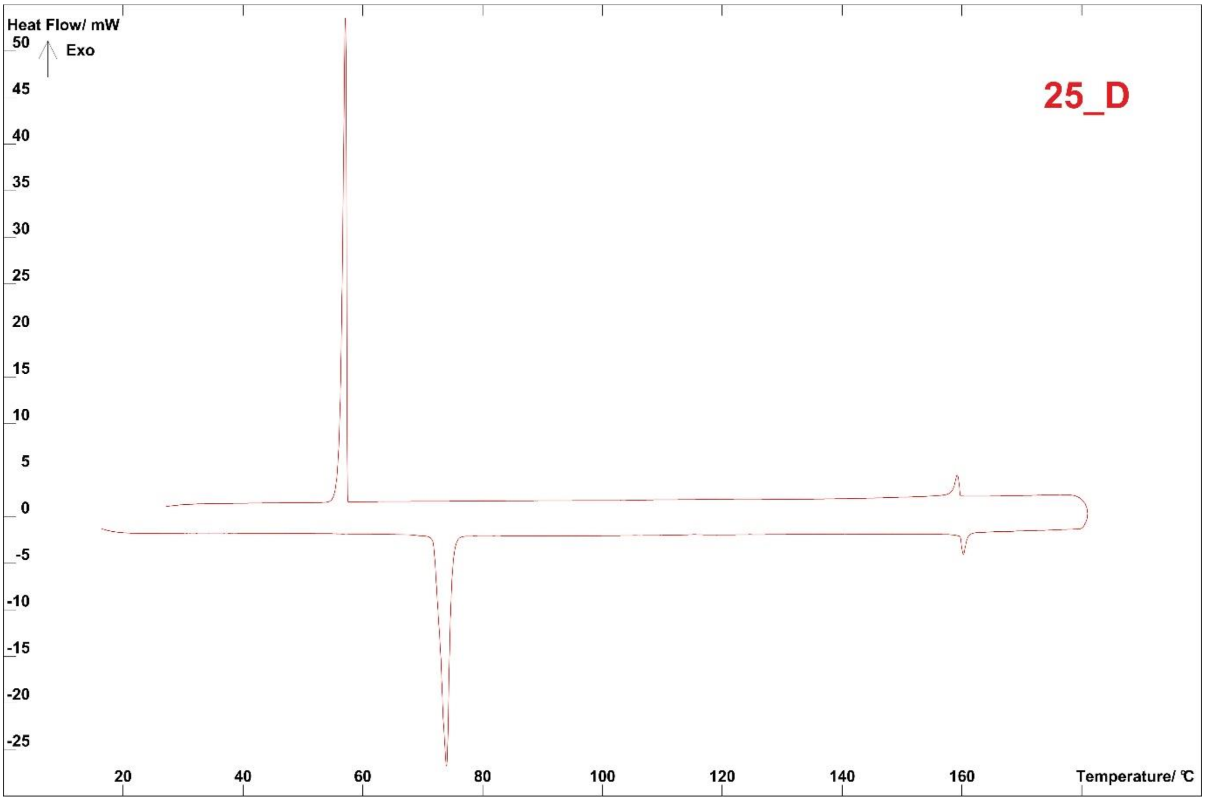

Figure S5. DSC spectrum of compound *25_D*.

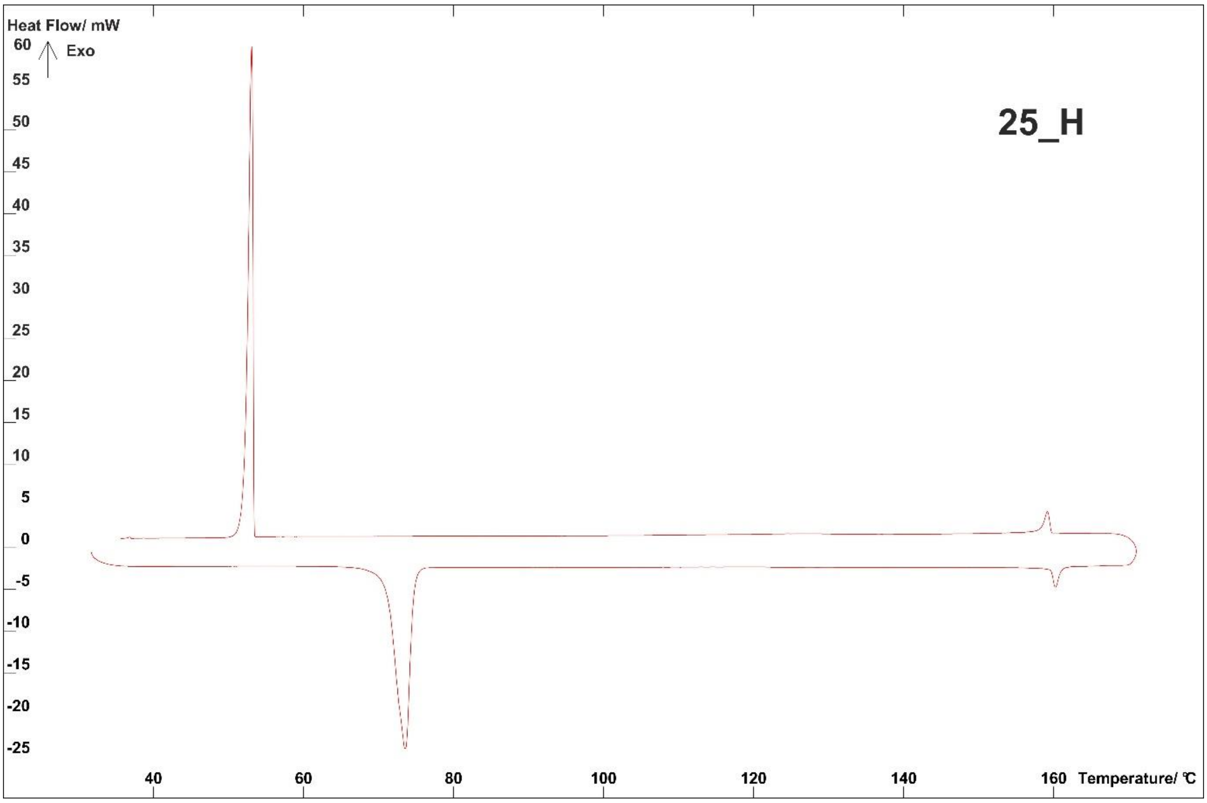

Figure S6. DSC spectrum of compound **25_H.**

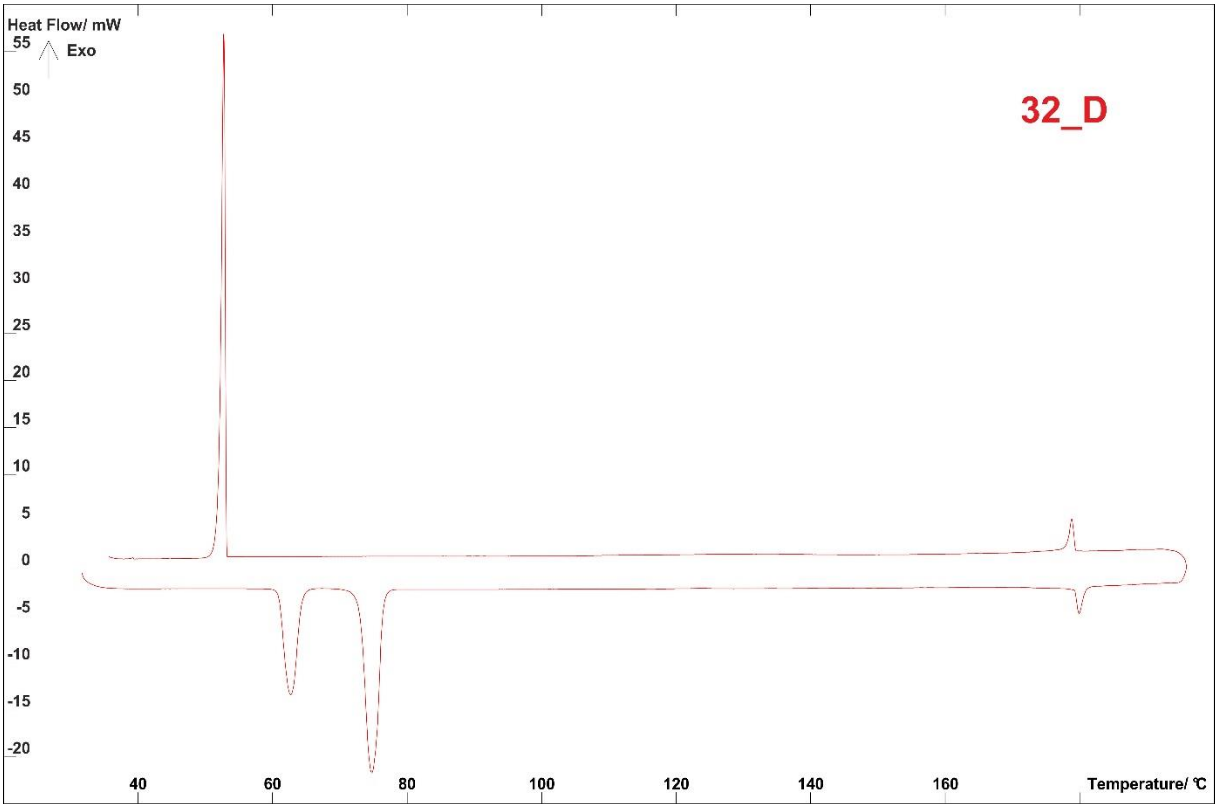

Figure S7. DSC spectrum of compound *32_D*.

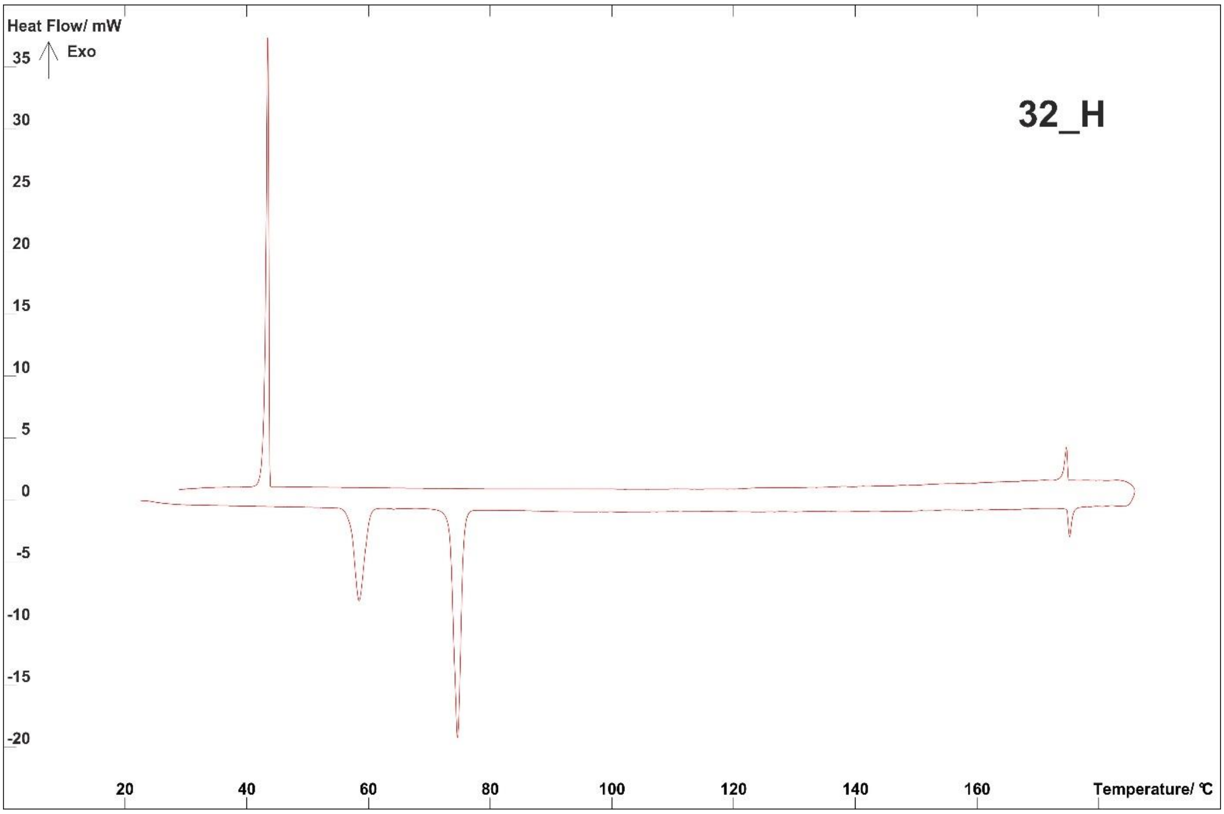

Figure S8. DSC spectrum of compound **32_H.**

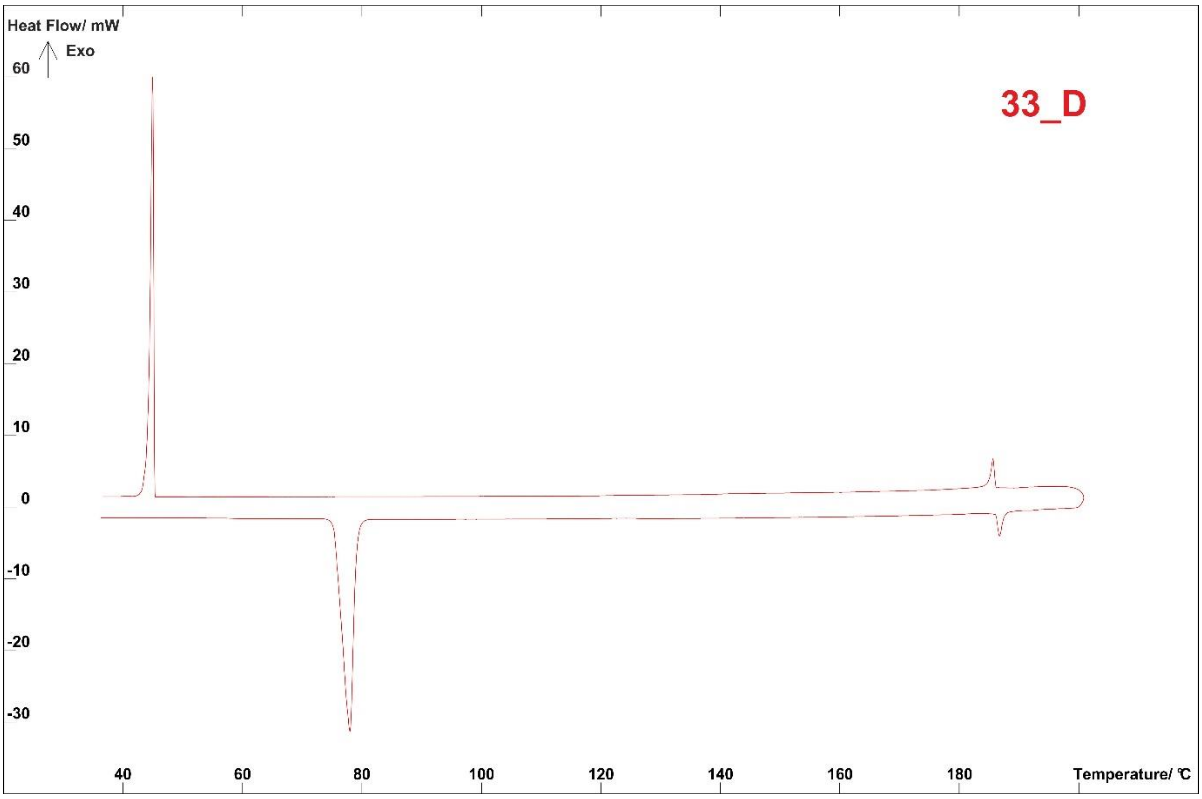

Figure S9. DSC spectrum of compound *33_D*.

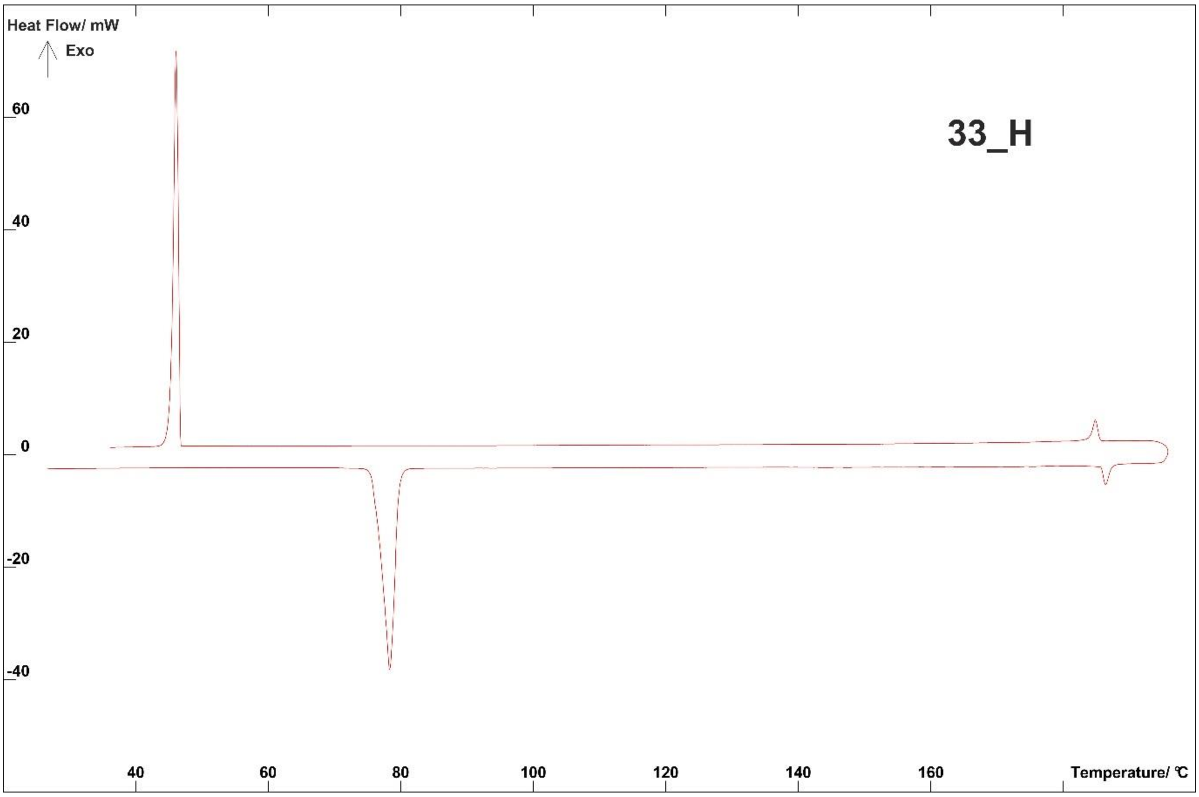

Figure S10. DSC spectrum of compound **33_H.**

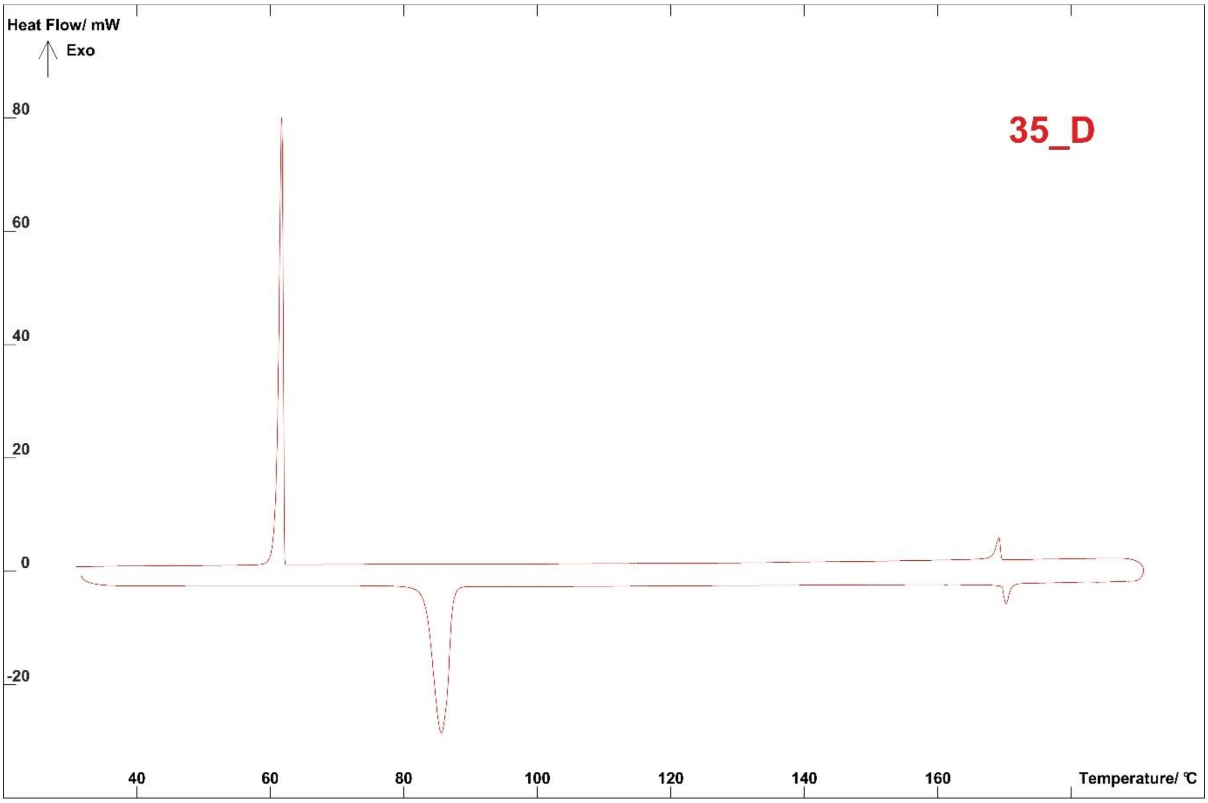

Figure S11. DSC spectrum of compound *35_D*.

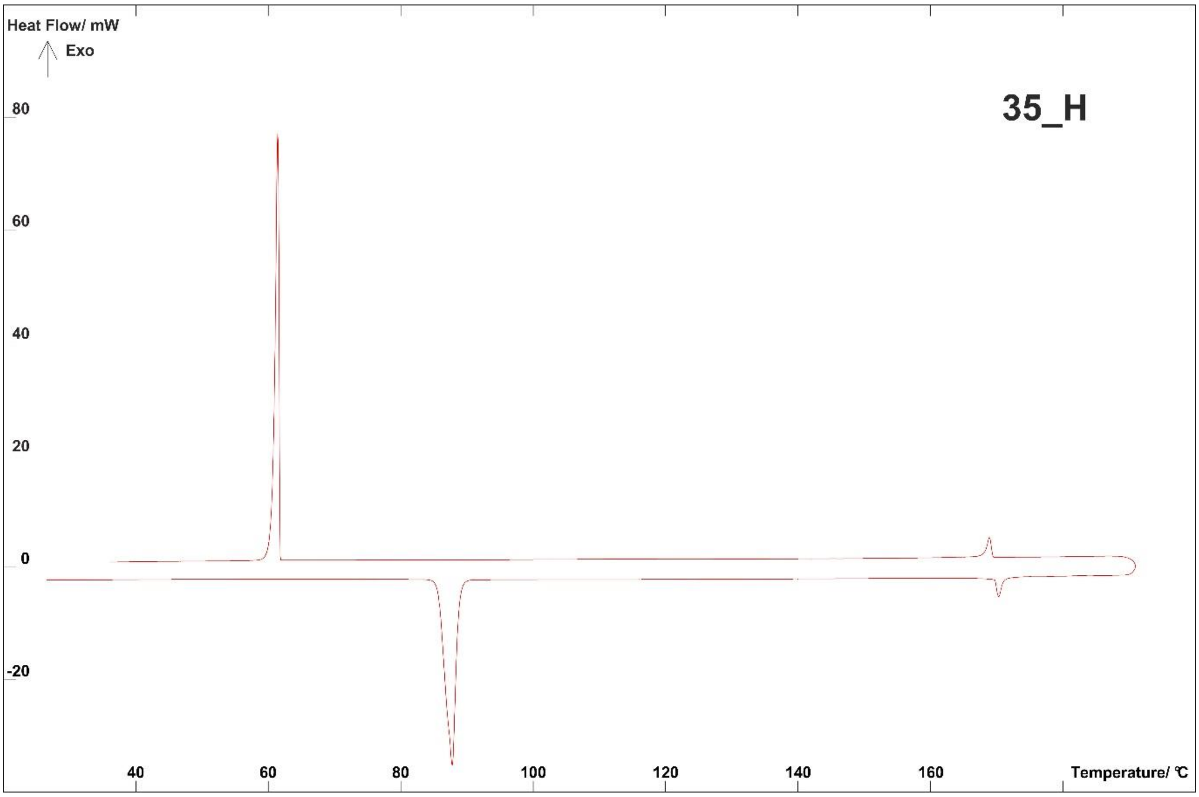

Figure S12. DSC spectrum of compound **35_H.**

Refractive Index Data

Table S1. Measured refractive indices (n_e and n_o) of *33_D* at λ = 443, 636, and 1550 nm, and at different temperatures.

T (°C)	λ (nm)					
	443 nm		636 nm		1550 nm	
	n_o	n_e	n_o	n_e	n_o	n_e
80	1.52876	1.90232	1.49755	1.83422	1.48077	1.76618
85	1.52849	1.89855	1.4977	1.83209	1.48094	1.76375
90	1.52832	1.89624	1.49663	1.82888	1.47882	1.76012
95	1.52722	1.89123	1.49588	1.82333	1.47691	1.75617
100	1.52713	1.88812	1.49543	1.81845	1.47517	1.75135
105	1.52654	1.88191	1.49499	1.81345	1.47559	1.74891
110	1.52644	1.87833	1.49321	1.80889	1.47652	1.74588
115	1.52566	1.87309	1.49243	1.80433	1.47489	1.74192
120	1.52509	1.86972	1.49201	1.79987	1.47438	1.73788
125	1.52497	1.86583	1.49163	1.79591	1.47367	1.73218
130	1.52522	1.8612	1.4911	1.78882	1.47429	1.72809
135	1.52501	1.85564	1.4915	1.78311	1.47519	1.72576
140	1.52467	1.84772	1.4905	1.77788	1.47338	1.71859
145	1.52459	1.84144	1.48999	1.77202	1.4719	1.71239
150	1.52434	1.83579	1.48992	1.76616	1.47173	1.70722
155	1.52501	1.82977	1.48891	1.75929	1.47197	1.70082
160	1.52524	1.82473	1.48992	1.75252	1.47128	1.69619
165	1.52498	1.81722	1.49002	1.74522	1.47078	1.6881
170	1.52596	1.80746	1.49046	1.73836	1.47056	1.68114
175	1.52611	1.79877	1.49178	1.73227	1.47188	1.67391
180	1.52771	1.78929	1.49321	1.72037	1.47244	1.66622
185	1.5287	1.77866	1.49453	1.70611	1.47378	1.65832
190	1.58672		1.56772		1.53372	
195	1.58348		1.56234		1.53134	

Table S2. Measured refractive indices (n_e and n_o) of **33_H** at λ = 443, 636, and 1550 nm, and at different temperatures.

T (°C)	λ (nm)					
	443 nm		636 nm		1550 nm	
	n_o	n_e	n_o	n_e	n_o	n_e
80	1.53101	1.90623	1.50021	1.83911	1.48355	1.7701
85	1.5319	1.90334	1.5005	1.83587	1.48388	1.76805
90	1.53211	1.90144	1.50075	1.83391	1.48326	1.76555
95	1.53101	1.89655	1.50033	1.82997	1.48021	1.76101
100	1.53081	1.89339	1.49988	1.8239	1.47828	1.75577
105	1.53009	1.88781	1.4999	1.81981	1.47688	1.75221
110	1.53001	1.88282	1.49811	1.81548	1.4772	1.74759
115	1.52799	1.87654	1.49777	1.80991	1.47592	1.74463
120	1.52822	1.87411	1.49622	1.80451	1.47532	1.74019
125	1.52774	1.86992	1.49601	1.80091	1.47522	1.73555
130	1.5292	1.86651	1.49651	1.79641	1.47424	1.73011
135	1.52718	1.85911	1.49622	1.78922	1.47688	1.72892
140	1.52725	1.85204	1.49599	1.78422	1.47521	1.722
145	1.52804	1.84681	1.49588	1.78011	1.47344	1.71609
150	1.52847	1.841	1.49506	1.77399	1.47172	1.70907
155	1.52804	1.83501	1.49322	1.76488	1.4726	1.70301
160	1.52818	1.82876	1.49563	1.75921	1.47321	1.70001
165	1.52899	1.8232	1.49572	1.75171	1.47273	1.69088
170	1.53003	1.81258	1.49681	1.74631	1.47221	1.68351
175	1.53015	1.8032	1.49772	1.73922	1.47362	1.67721
180	1.53104	1.79344	1.49821	1.72691	1.47592	1.67059
185	1.53326	1.78371	1.49901	1.71166	1.47812	1.66391
190	1.58722		1.56882		1.53488	
195	1.58455		1.58458		1.53344	

Table S3. Measured refractive indices (n_e and n_o) of *23_D* at λ = 443, 636, and 1550 nm, and at different temperatures.

T (°C)	λ (nm)					
	443 nm		636 nm		1550 nm	
	n_o	n_e	n_o	n_e	n_o	n_e
70	1.52888	1.91471	1.49848	1.85481	1.47848	1.78181
75	1.52876	1.91322	1.49826	1.85312	1.47826	1.78012
80	1.52781	1.91071	1.49751	1.85081	1.47651	1.77781
85	1.52788	1.90851	1.49768	1.84861	1.47668	1.77561
90	1.52791	1.90638	1.49741	1.84648	1.47541	1.77248
95	1.52812	1.9012	1.49812	1.84179	1.47412	1.76729
100	1.52789	1.89877	1.49769	1.83887	1.47369	1.76487
105	1.52723	1.89342	1.49713	1.83352	1.47313	1.76052
110	1.52667	1.89001	1.49657	1.83011	1.47357	1.75771
115	1.52599	1.88507	1.49569	1.82517	1.47269	1.75317
120	1.52552	1.88123	1.49532	1.82133	1.47232	1.75103
125	1.52592	1.87771	1.49572	1.81781	1.47272	1.74681
130	1.52489	1.87133	1.49479	1.81143	1.47179	1.74043
135	1.52581	1.86638	1.49561	1.80648	1.47261	1.73648
140	1.52591	1.86129	1.49561	1.80139	1.47261	1.733
145	1.52533	1.85571	1.49533	1.79581	1.47333	1.72781
150	1.52601	1.85003	1.49611	1.79013	1.47311	1.72313
155	1.52612	1.8451	1.49602	1.78519	1.47302	1.71719
160	1.52655	1.83801	1.49645	1.77811	1.47345	1.71211
165	1.52669	1.83111	1.49659	1.77121	1.47359	1.70621
170	1.58466		1.56721		1.53444	
175	1.58328		1.56549		1.53328	

Table S4. Measured refractive indices (n_e and n_o) of **23_H** at λ = 443, 636, and 1550 nm, and at different temperatures.

T (°C)	λ (nm)					
	443 nm		636 nm		1550 nm	
	n_o	n_e	n_o	n_e	n_o	n_e
70	1.52918	1.91671	1.49982	1.85681	1.47948	1.78381
75	1.52926	1.91522	1.49959	1.85512	1.47996	1.78212
80	1.52821	1.91211	1.49883	1.85381	1.47851	1.78081
85	1.52828	1.90991	1.49899	1.85031	1.47868	1.77861
90	1.52821	1.90768	1.49882	1.84818	1.47841	1.77558
95	1.52832	1.9022	1.4994	1.84429	1.47812	1.77209
100	1.528	1.89987	1.49891	1.84147	1.47869	1.76977
105	1.52903	1.89542	1.49849	1.83662	1.47813	1.76652
110	1.52867	1.89301	1.49781	1.83451	1.47757	1.76211
115	1.52801	1.88787	1.49692	1.82827	1.47769	1.75897
120	1.52752	1.88393	1.49649	1.82443	1.47732	1.75693
125	1.52803	1.87991	1.49688	1.81981	1.47672	1.75081
130	1.5251	1.87333	1.49598	1.81343	1.47679	1.74643
135	1.52881	1.86988	1.49691	1.80818	1.47761	1.74248
140	1.52799	1.86429	1.4969	1.80339	1.47761	1.73903
145	1.52713	1.85811	1.49655	1.79801	1.47633	1.73181
150	1.52781	1.85343	1.49741	1.79393	1.47611	1.72613
155	1.52802	1.84831	1.49742	1.78909	1.47602	1.72119
160	1.52855	1.84101	1.49765	1.77991	1.47545	1.71301
165	1.52839	1.83311	1.49791	1.77321	1.4759	1.70991
170	1.58588		1.56882		1.53572	
175	1.58482		1.56622		1.53355	

Table S5. Measured refractive indices (n_e and n_o) of *25_D* at λ = 443, 636, and 1550 nm, and at different temperatures.

T (°C)	λ (nm)					
	443 nm		636 nm		1550 nm	
	n_o	n_e	n_o	n_e	n_o	n_e
75	1.52855	1.89111	1.49875	1.82111	1.47975	1.75811
80	1.52881	1.89018	1.49871	1.81818	1.47981	1.75618
85	1.52781	1.88622	1.49781	1.81522	1.47891	1.75322
90	1.52801	1.88278	1.49821	1.81278	1.47821	1.75078
95	1.52811	1.87961	1.49811	1.80961	1.47811	1.74761
100	1.52798	1.87579	1.49808	1.80579	1.47808	1.74479
105	1.528	1.87129	1.4981	1.80129	1.4781	1.74229
110	1.5278	1.8659	1.49782	1.7959	1.47782	1.7379
115	1.52755	1.86101	1.49765	1.7911	1.47765	1.73411
120	1.52677	1.85512	1.49667	1.78562	1.47667	1.72862
125	1.52666	1.85151	1.49676	1.78101	1.47676	1.72301
130	1.52601	1.84521	1.49571	1.77461	1.47571	1.71761
135	1.52632	1.84002	1.49632	1.77002	1.47632	1.71202
140	1.52644	1.83403	1.49664	1.76403	1.47664	1.70603
145	1.527	1.82844	1.49722	1.75844	1.47722	1.70044
150	1.52711	1.82001	1.49731	1.75001	1.47731	1.69501
155	1.52776	1.8108	1.49796	1.7408	1.47796	1.68708
160	1.57988		1.56599		1.52899	
165	1.57766		1.56328		1.52778	

Table S6. Measured refractive indices (n_e and n_o) of **25_H** at λ = 443, 636, and 1550 nm, and at different temperatures.

T (°C)	λ (nm)					
	443 nm		636 nm		1550 nm	
	n_o	n_e	n_o	n_e	n_o	n_e
75	1.5291	1.89222	1.49995	1.82331	1.48115	1.76101
80	1.52912	1.89122	1.49991	1.81998	1.48101	1.75848
85	1.5288	1.888	1.49981	1.81772	1.48101	1.75552
90	1.52866	1.88411	1.50021	1.81548	1.48001	1.75358
95	1.52901	1.88111	1.50011	1.81199	1.47991	1.74981
100	1.5285	1.87772	1.50008	1.80869	1.47988	1.74749
105	1.52911	1.87391	1.5001	1.80359	1.47982	1.74569
110	1.5291	1.86877	1.49992	1.79889	1.47942	1.73988
115	1.52901	1.86383	1.49975	1.7945	1.47935	1.73681
120	1.5288	1.85788	1.49887	1.78892	1.47837	1.73002
125	1.52911	1.85492	1.49876	1.78431	1.47876	1.72581
130	1.52881	1.84891	1.49801	1.77691	1.47751	1.71991
135	1.5281	1.84201	1.49902	1.77512	1.47822	1.71542
140	1.52855	1.83699	1.49874	1.76743	1.47834	1.70893
145	1.52888	1.83101	1.49972	1.76194	1.47902	1.70324
150	1.52901	1.8229	1.49981	1.75291	1.47911	1.69751
155	1.52942	1.81377	1.50096	1.7443	1.47996	1.68988
160	1.58002		1.56732		1.52981	
165	1.57891		1.56482		1.52822	

Mass Spectrum (MS) Data

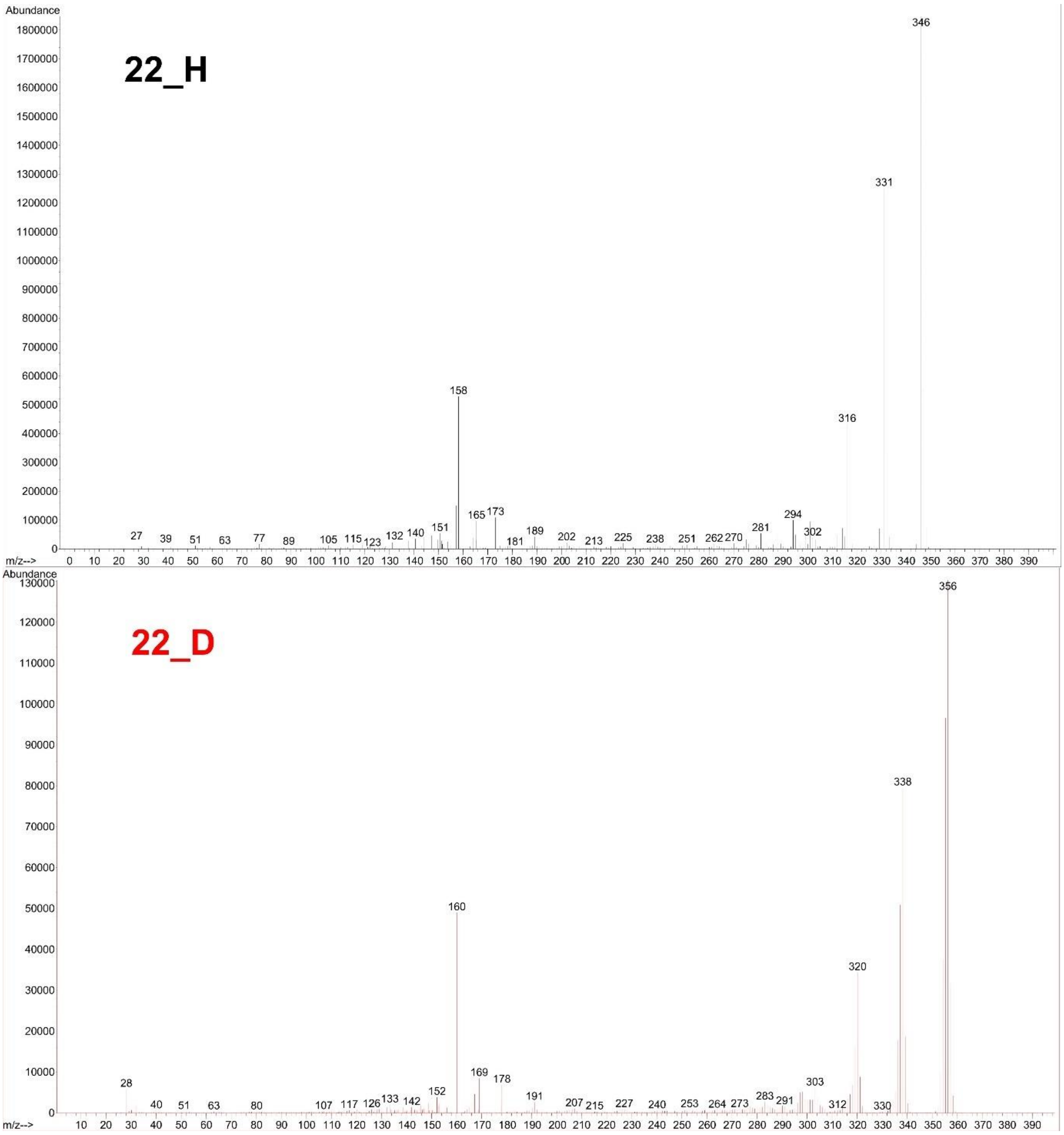

Figure S13. MS spectrum of compounds **22_H** and *22_D*.

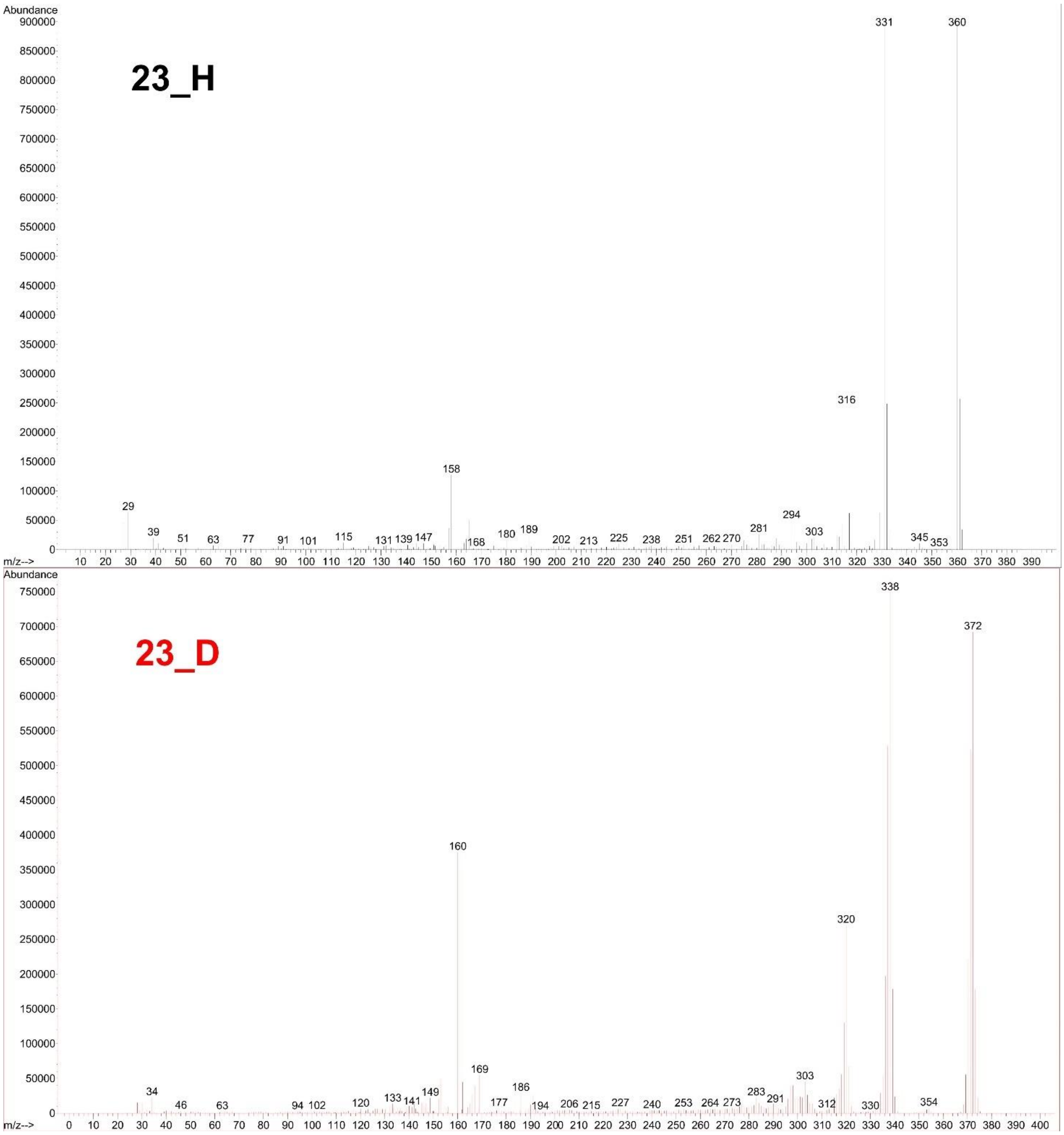

Figure S14. MS spectrum of compounds **23_H** and *23_D*.

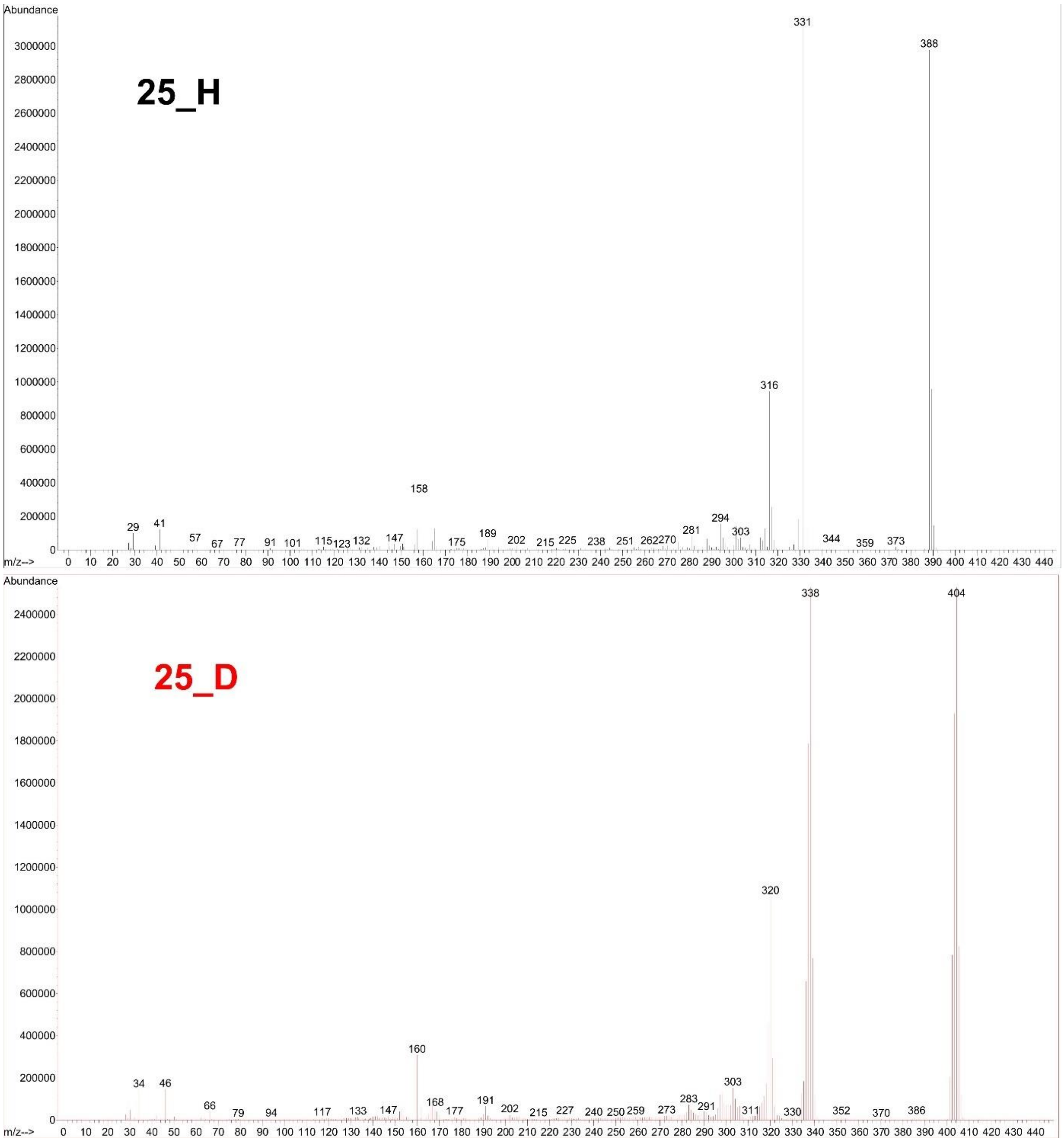

Figure S15. MS spectrum of compounds **25_H** and *25_D*.

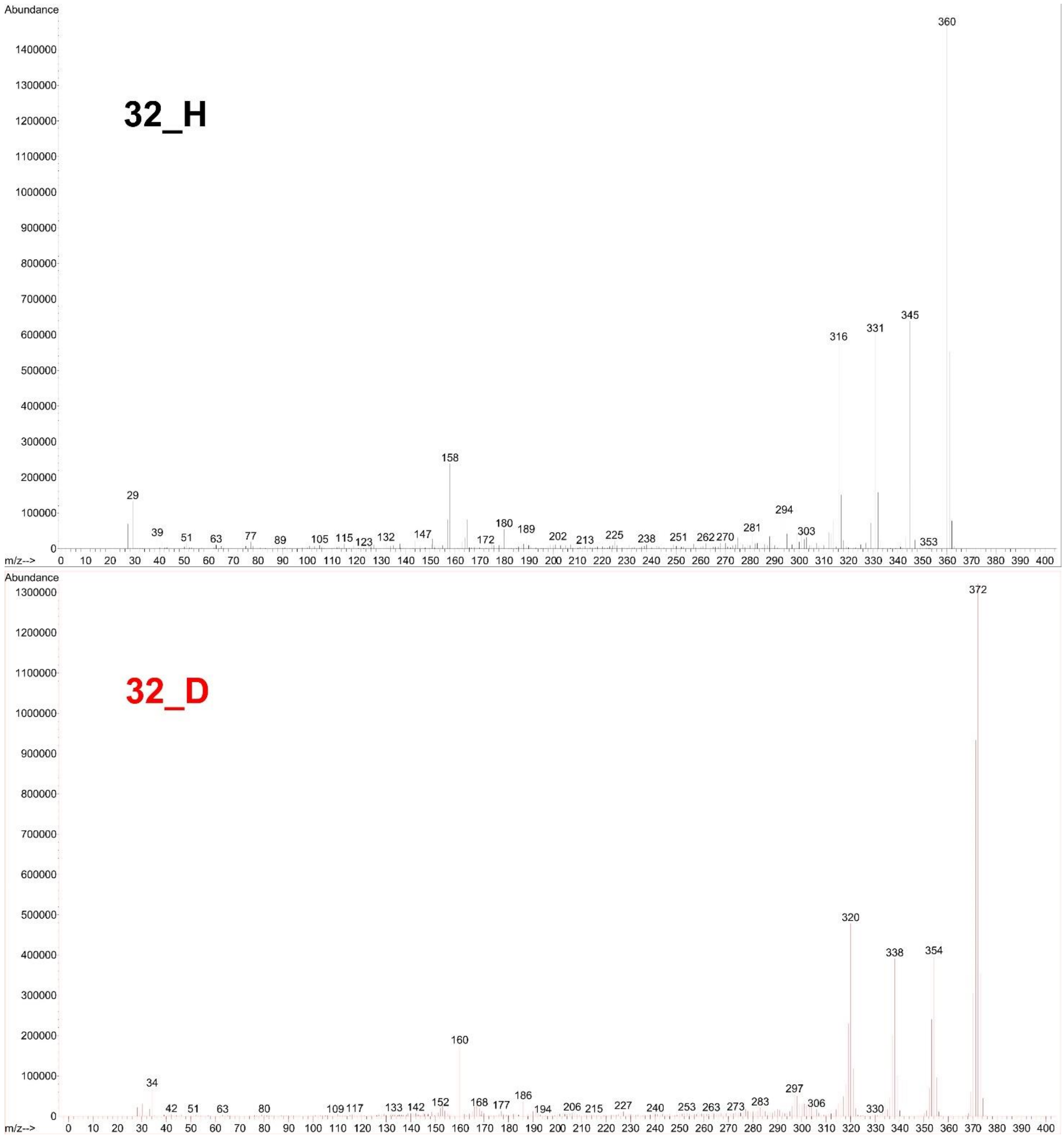

Figure S16. MS spectrum of compounds **32_H** and *32_D*.

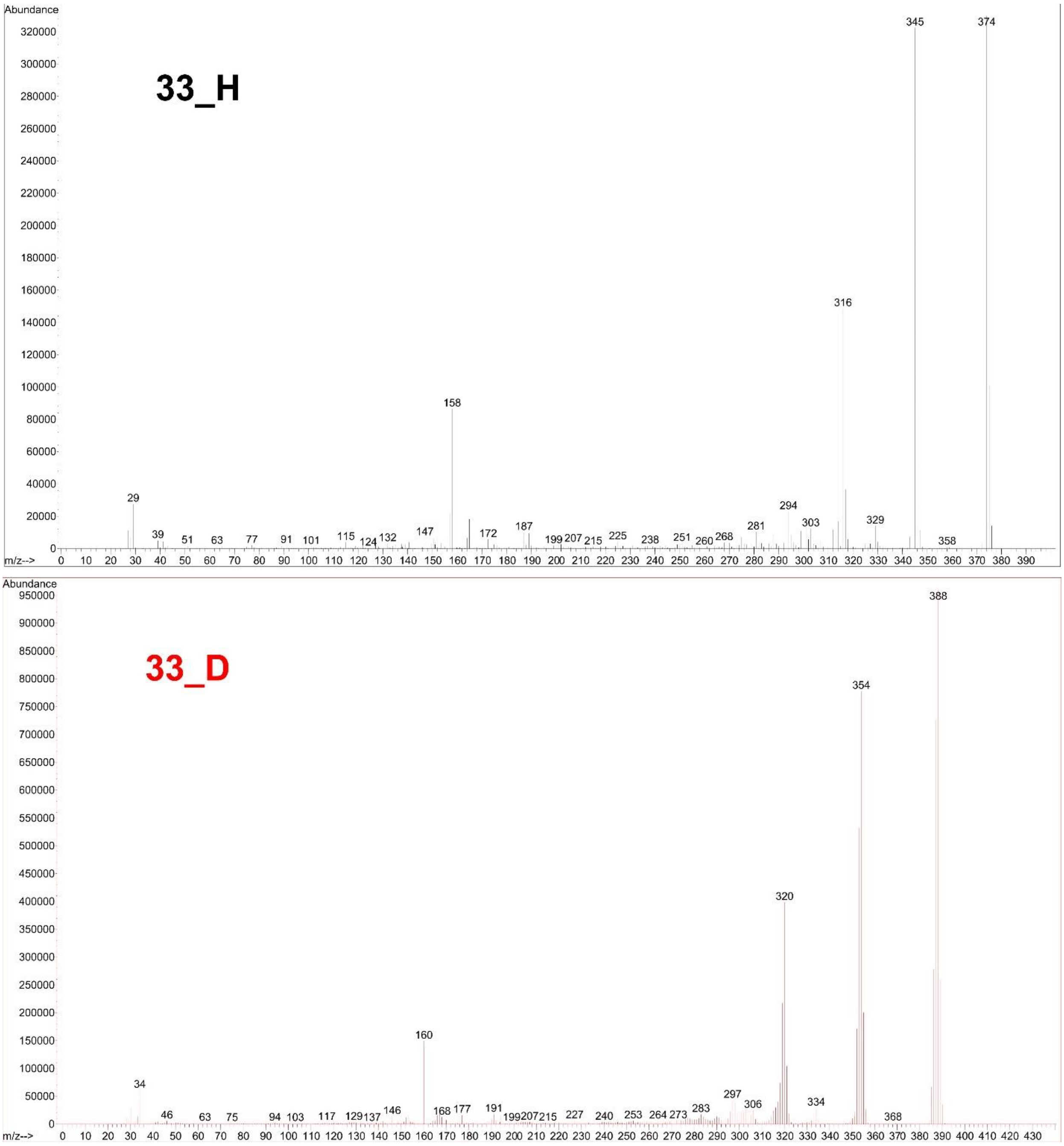

Figure S17. MS spectrum of compounds **33_H** and *33_D*.

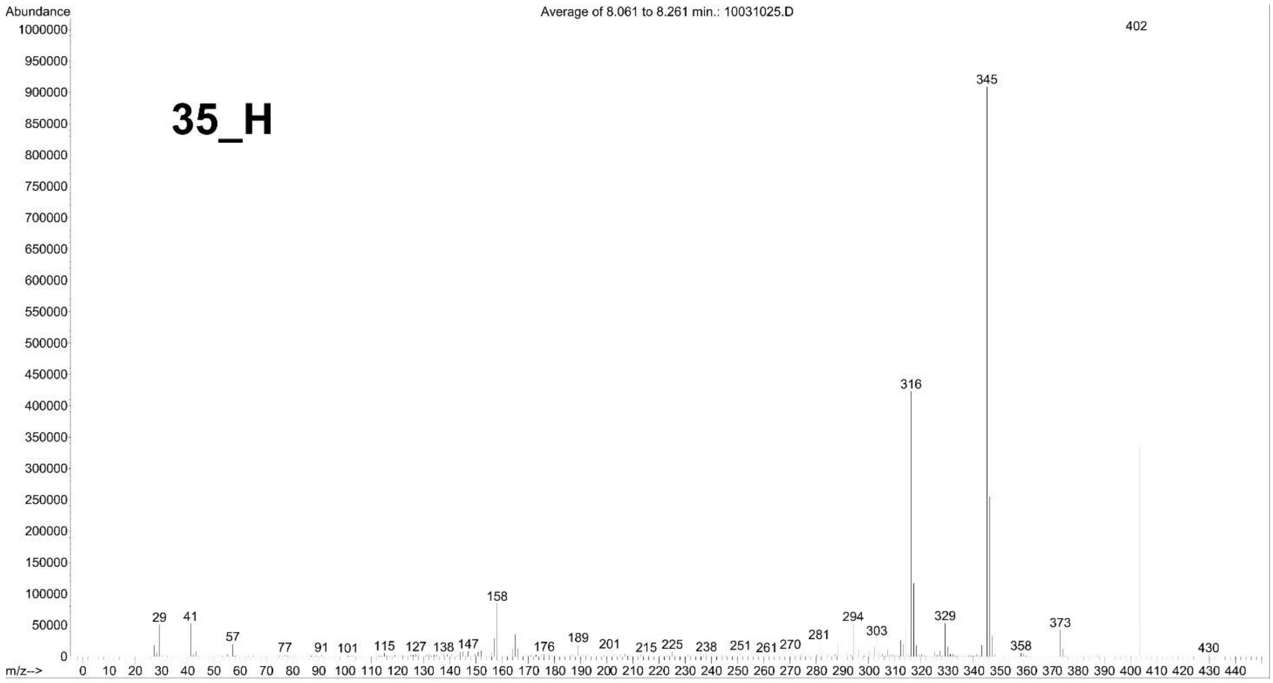

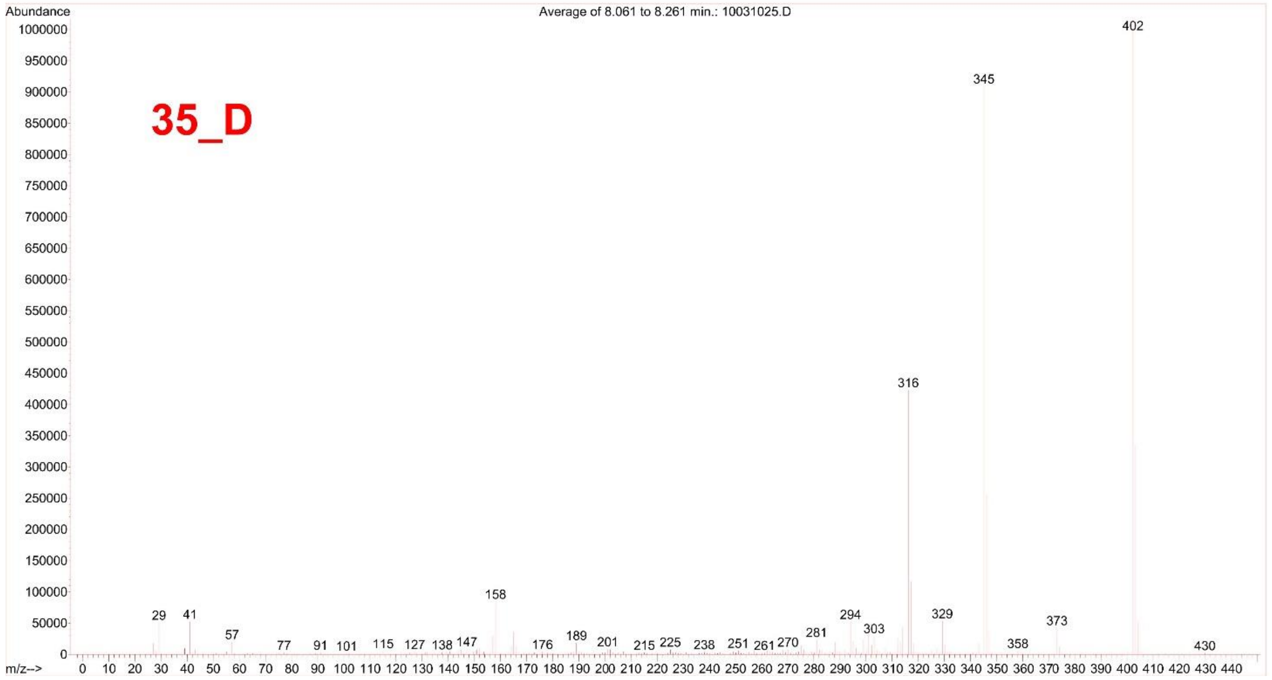

Figure S18. MS spectrum of compounds **35_H** and *35_D*.

Medium Wavelength Infrared (MWIR) Data

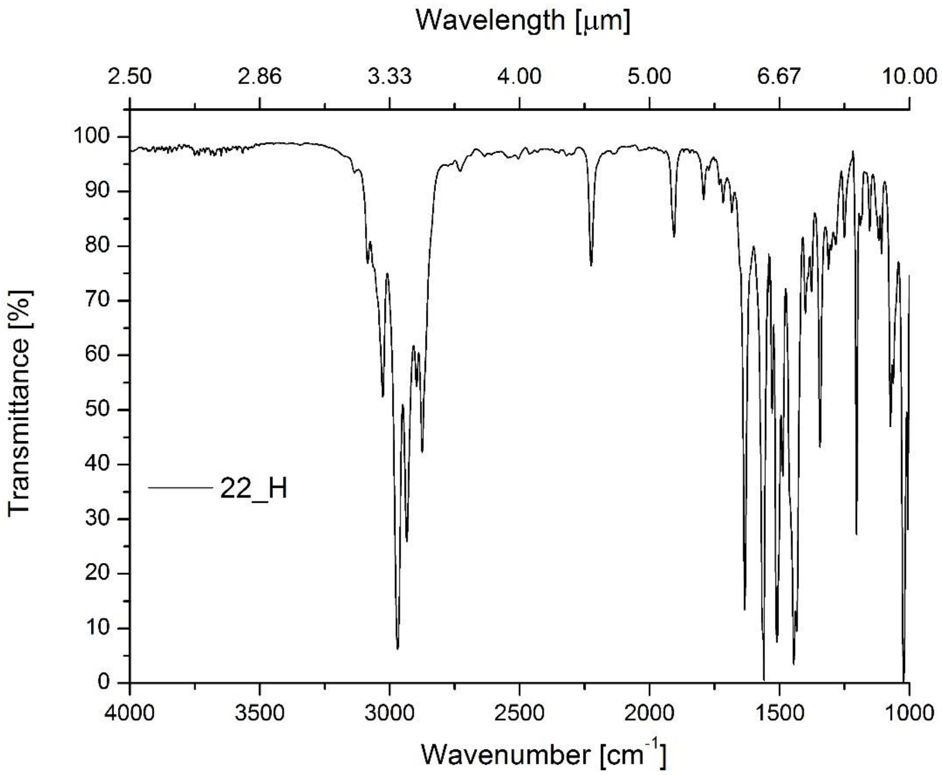

Figure S19. IR spectrum of compound **22_H**.

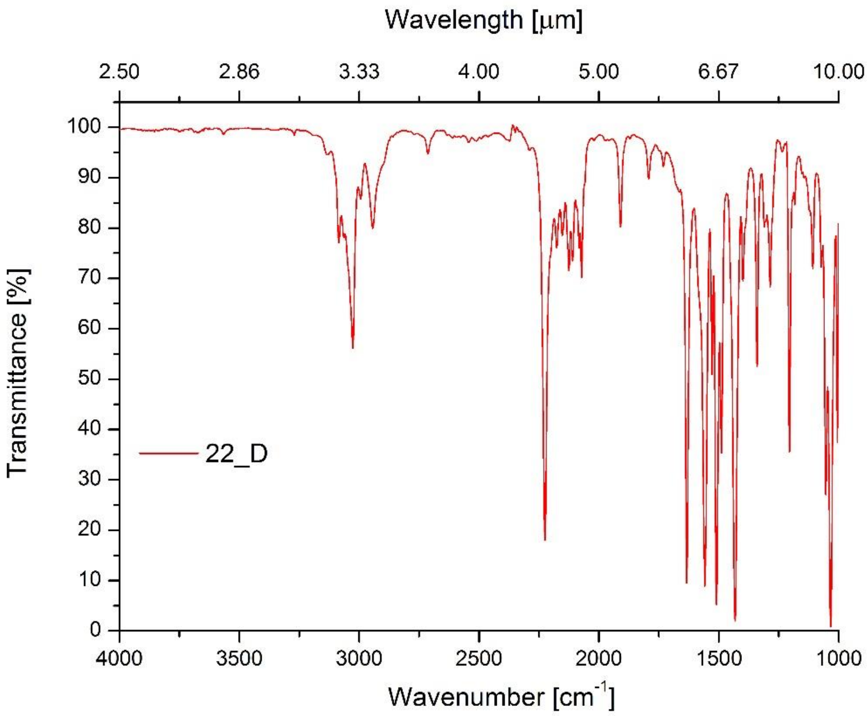

Figure S20. IR spectrum of compound 22_*D*.

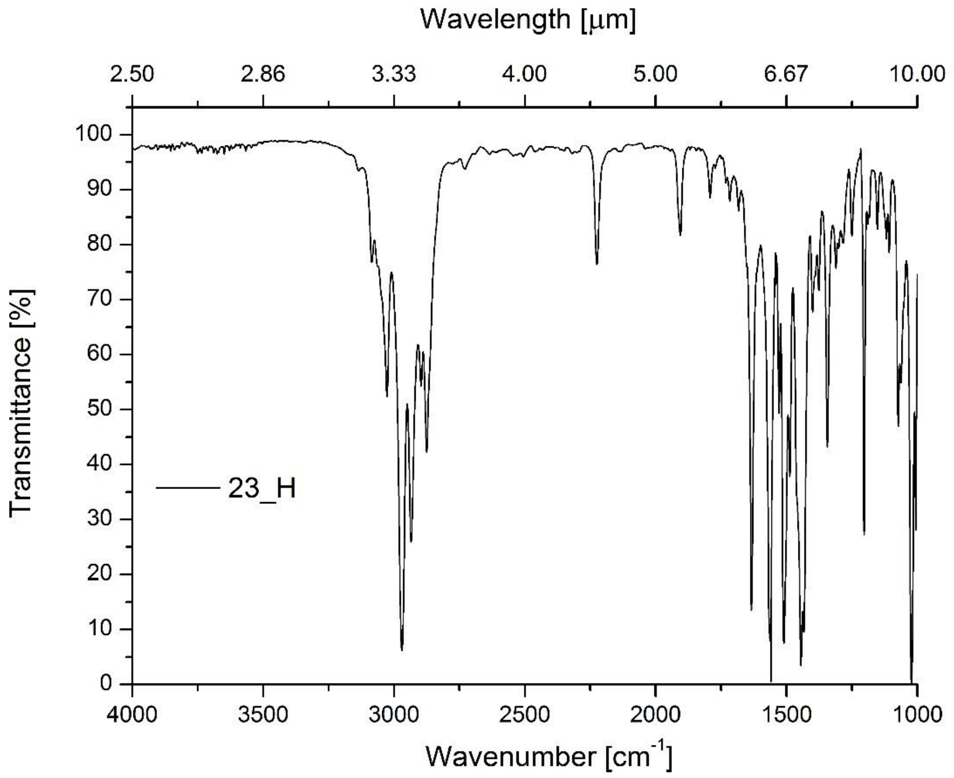

Figure S21. IR spectrum of compound **23_H.**

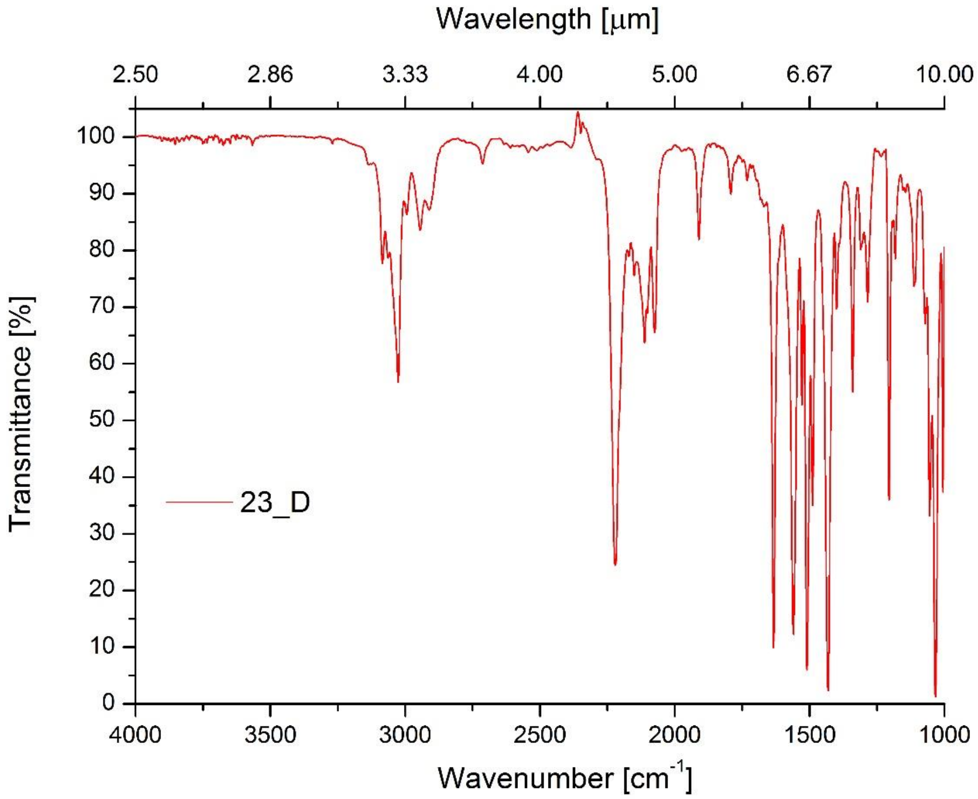

Figure S22. IR spectrum of compound *23_D*.

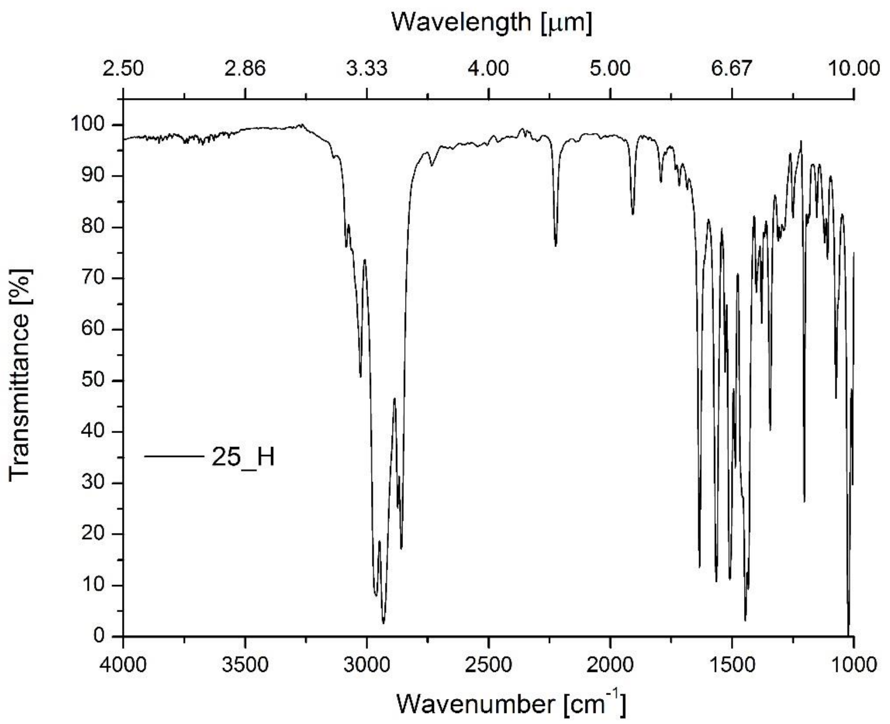

Figure S23. IR spectrum of compound **25_H**.

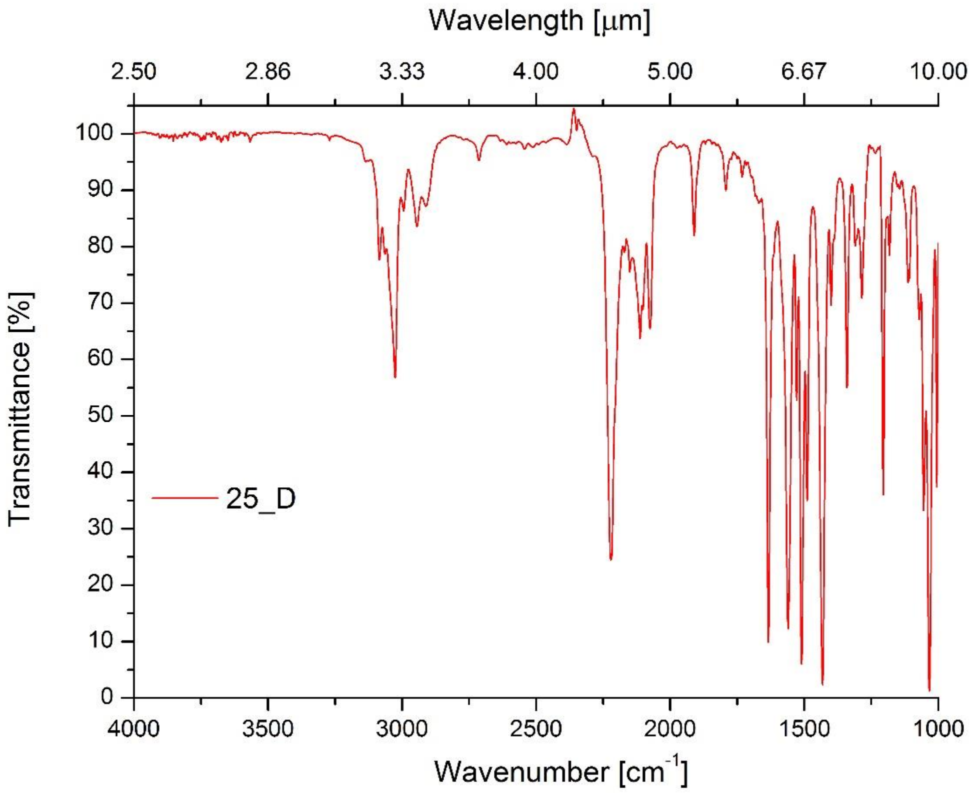

Figure S24. IR spectrum of compound ***25_D***.

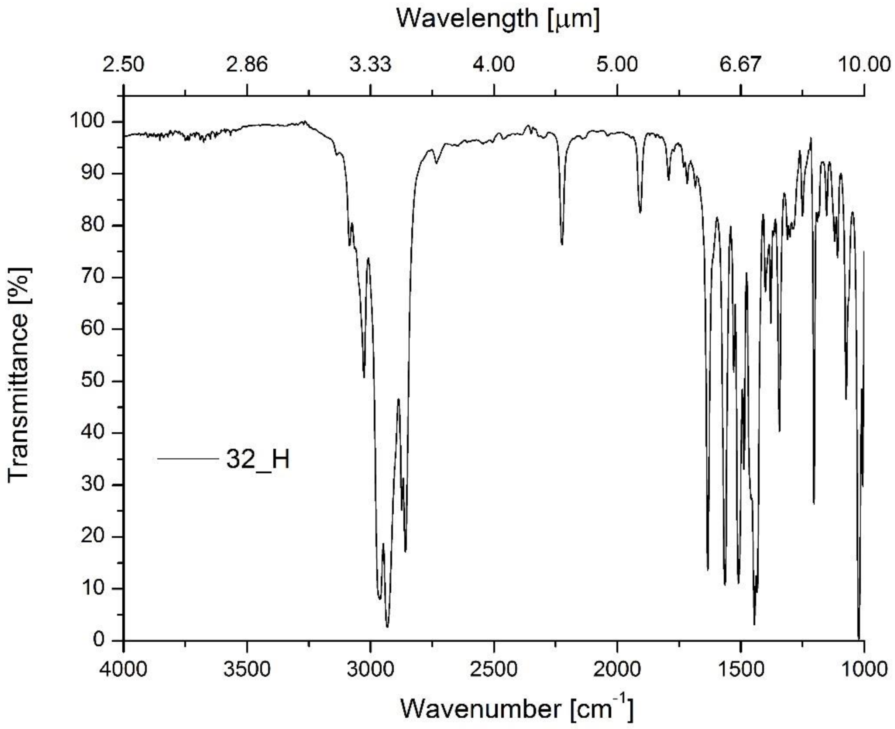

Figure S25. IR spectrum of compound **32_H.**

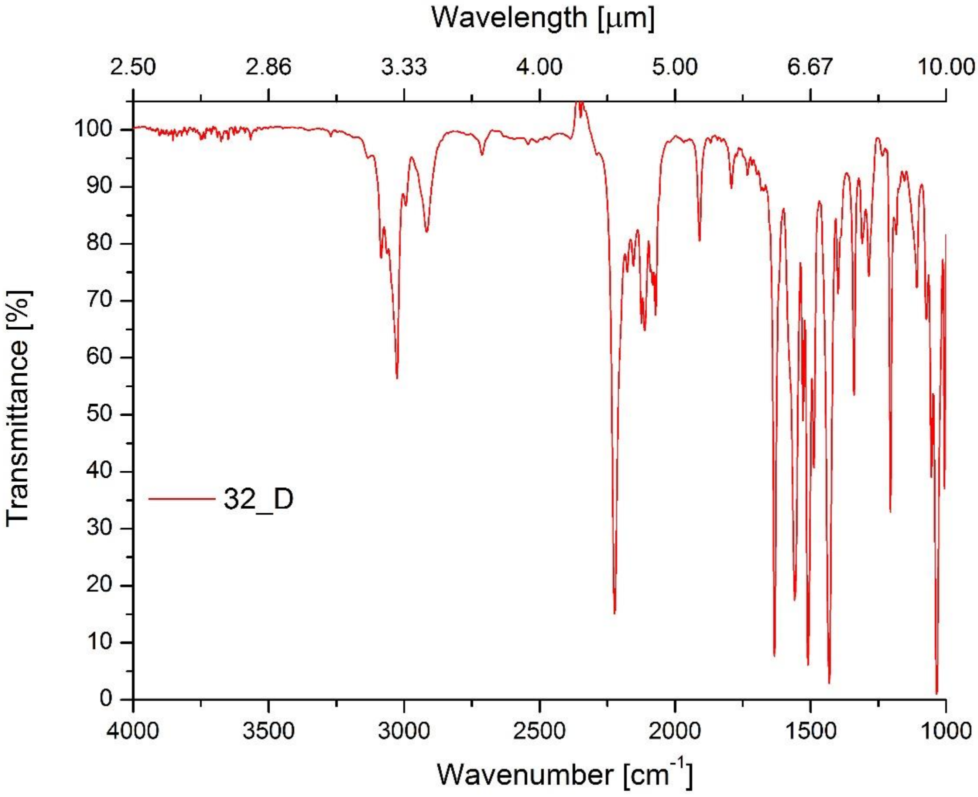

Figure S26. IR spectrum of compound *32_D*.

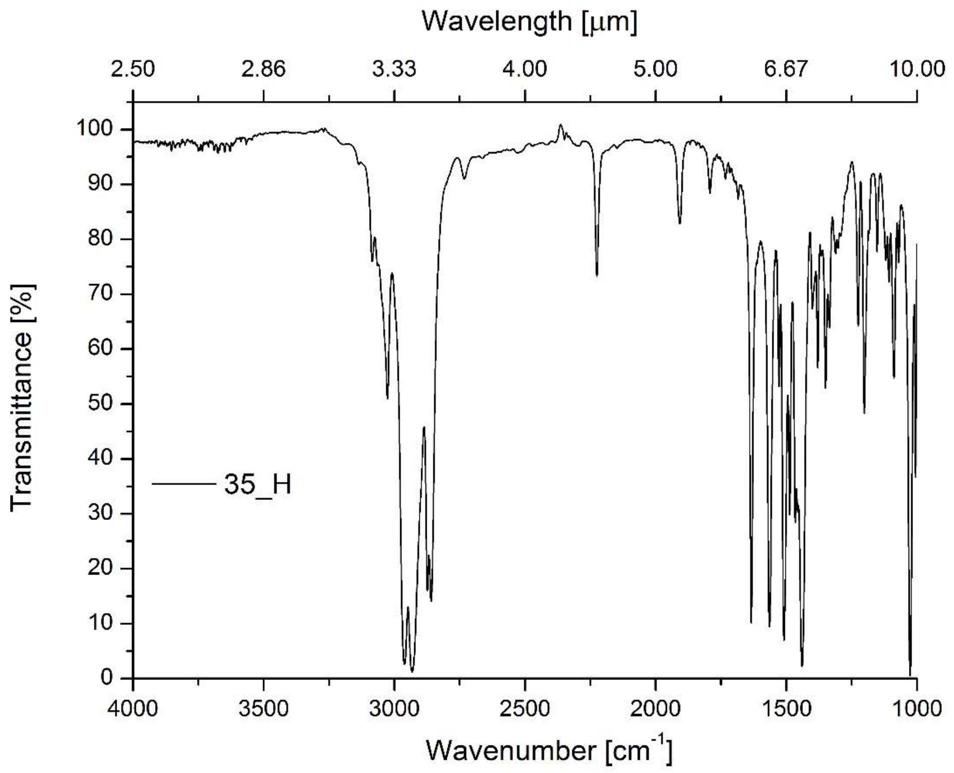

Figure S27. IR spectrum of compound **35_H**.

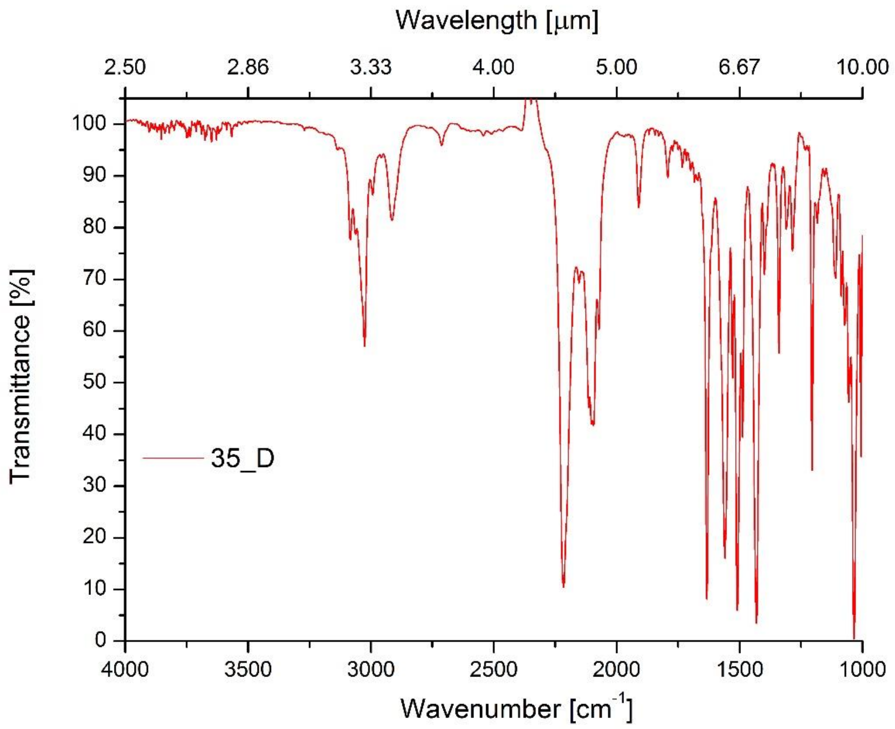

Figure S28. IR spectrum of compound ***35_D***.

Nuclear Magnetic Resonance (NMR) Data

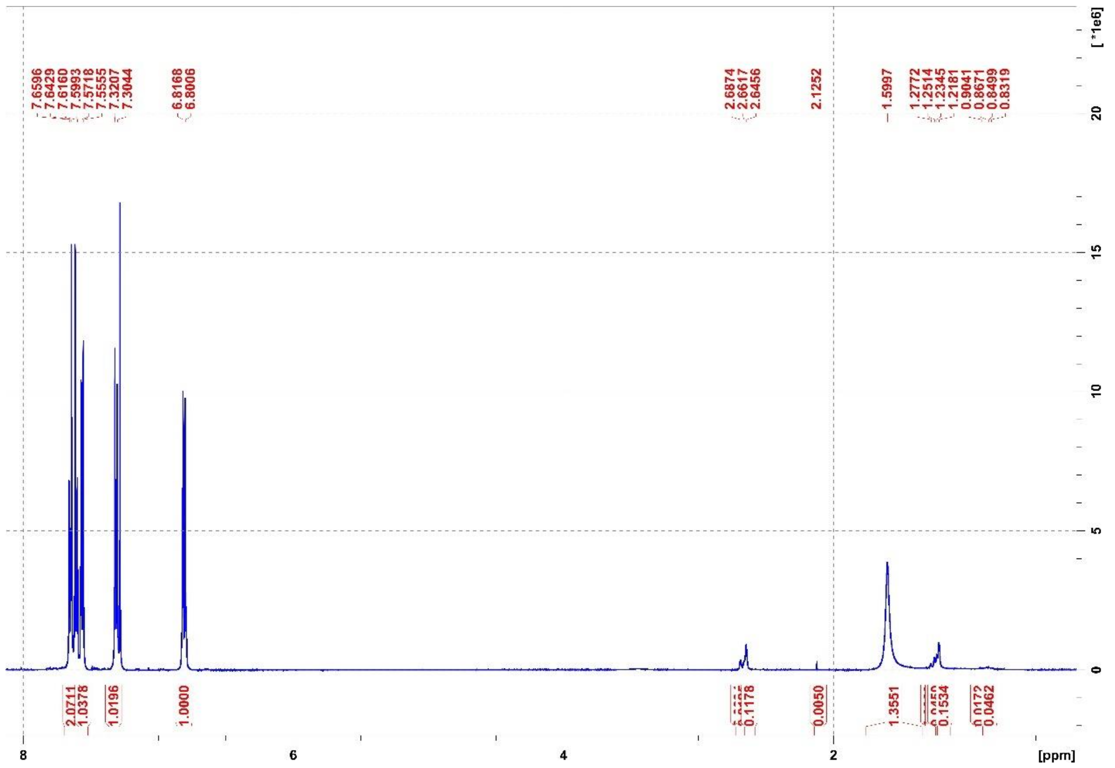

Figure S29. ^{1}H NMR spectrum of compound 22_D.

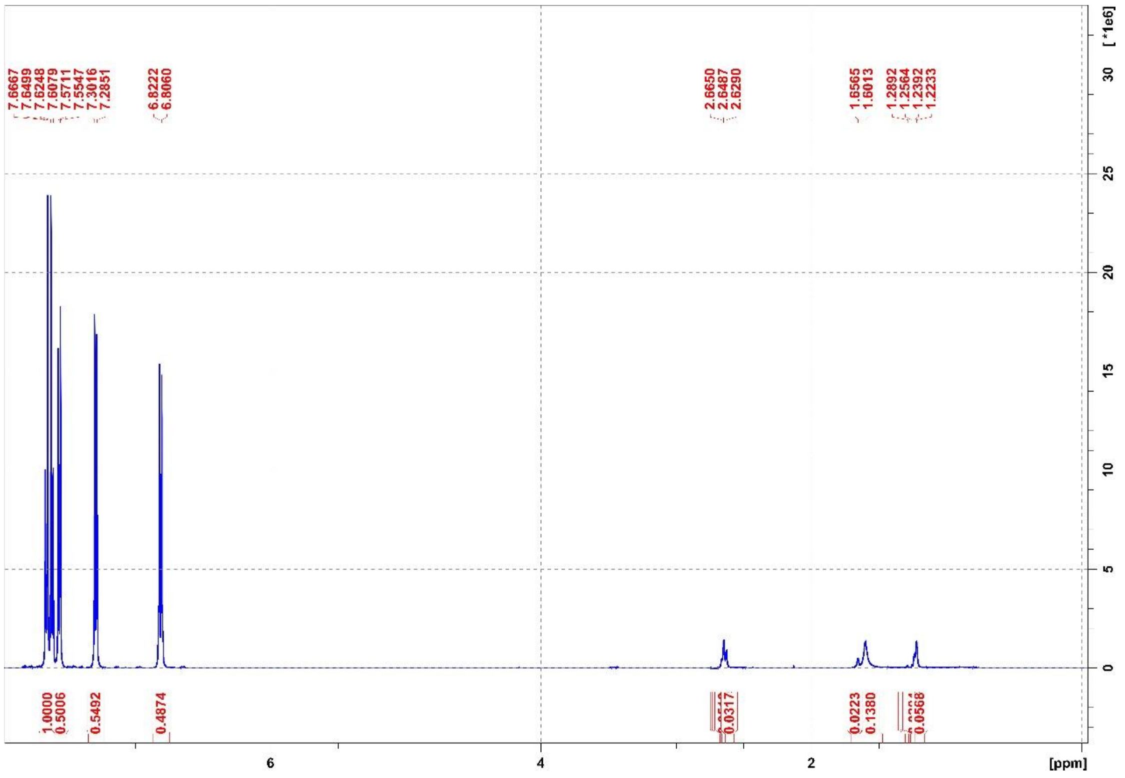

Figure S30. ^{1}H NMR spectrum of compound 23_D.

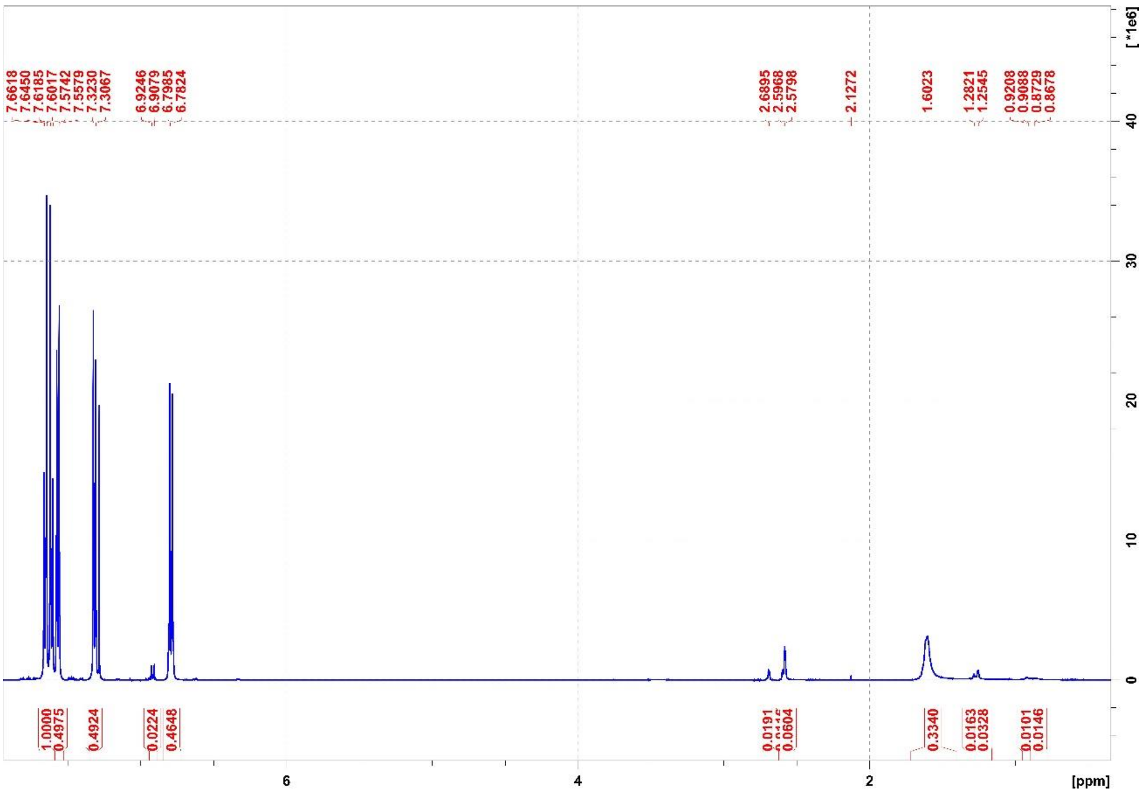

Figure S31. ^{1}H NMR spectrum of compound *32_D*.

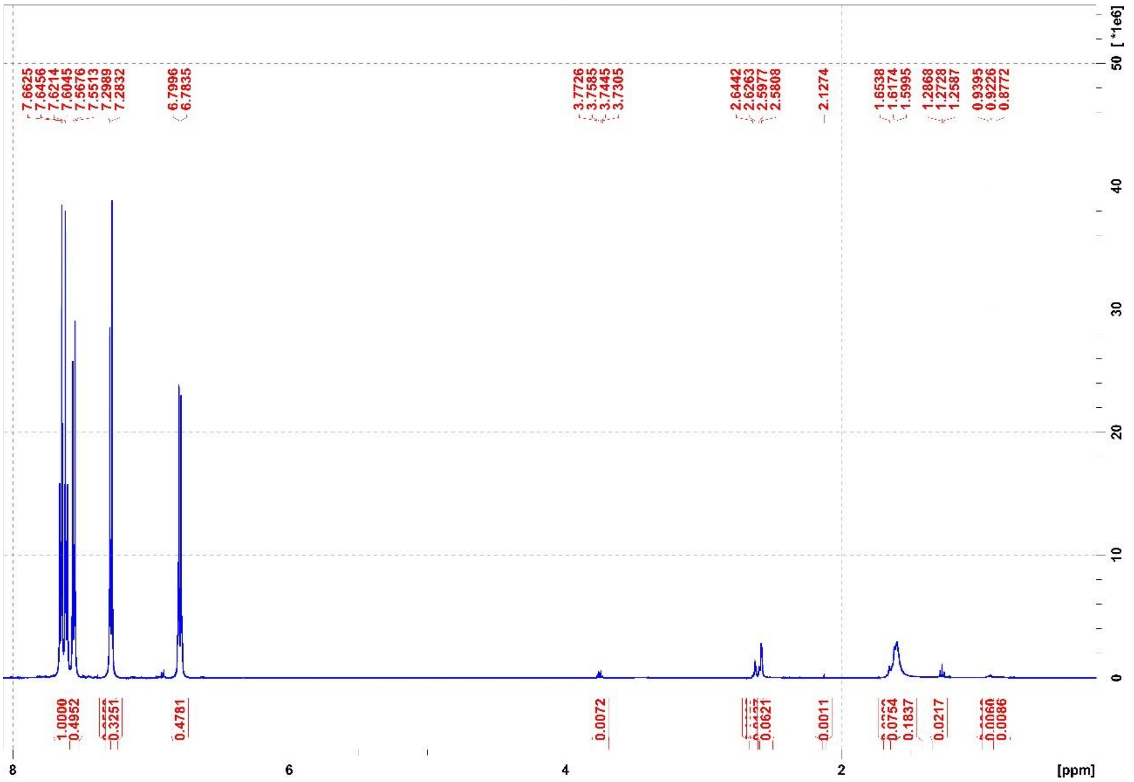

Figure S32. ^{1}H NMR spectrum of compound *33_D*.

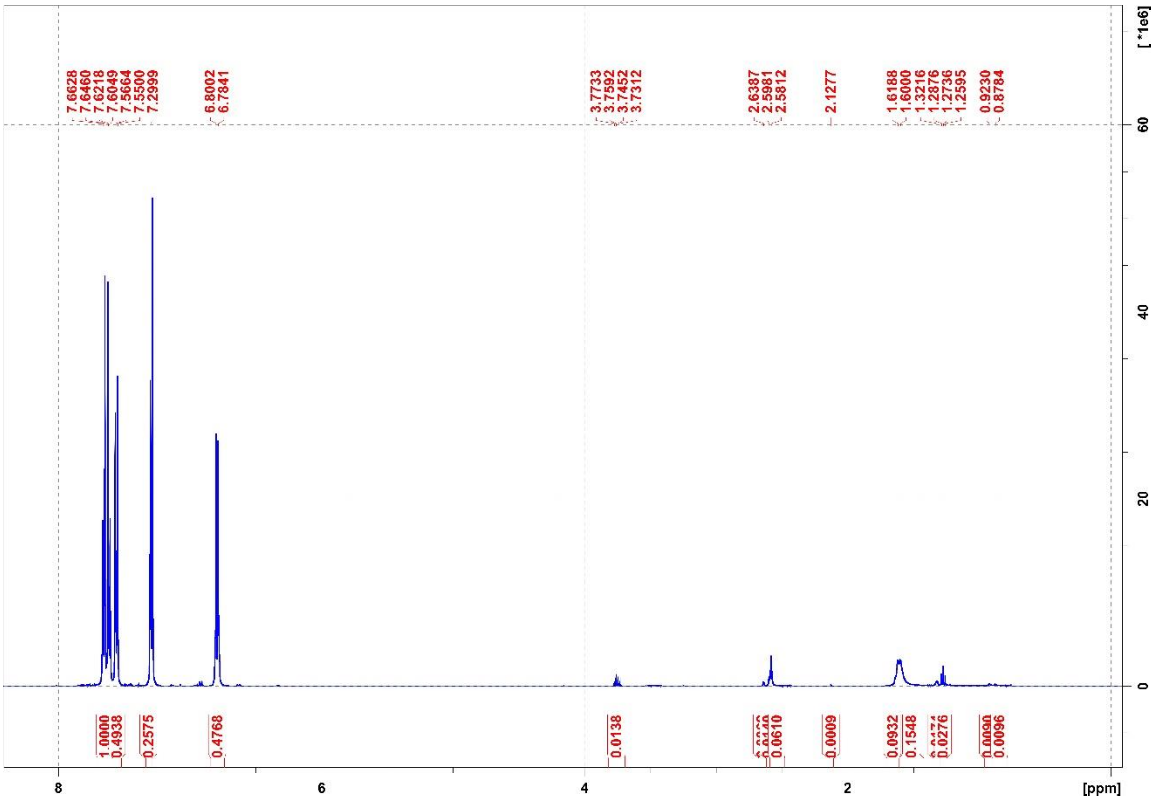

Figure S33. ^{1}H NMR spectrum of compound *35_D*.

Polarising Optical Microscope (POM) textures

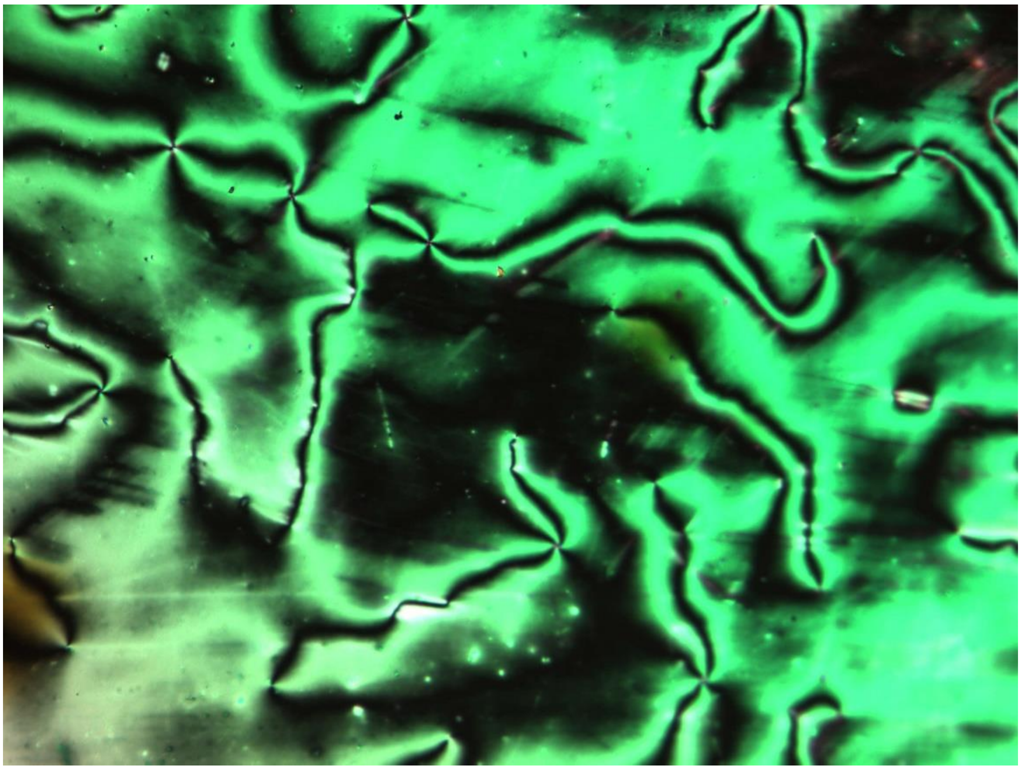

Figure S34. POM texture of nematic phase of compound *33_D* (T = 150.0°C).

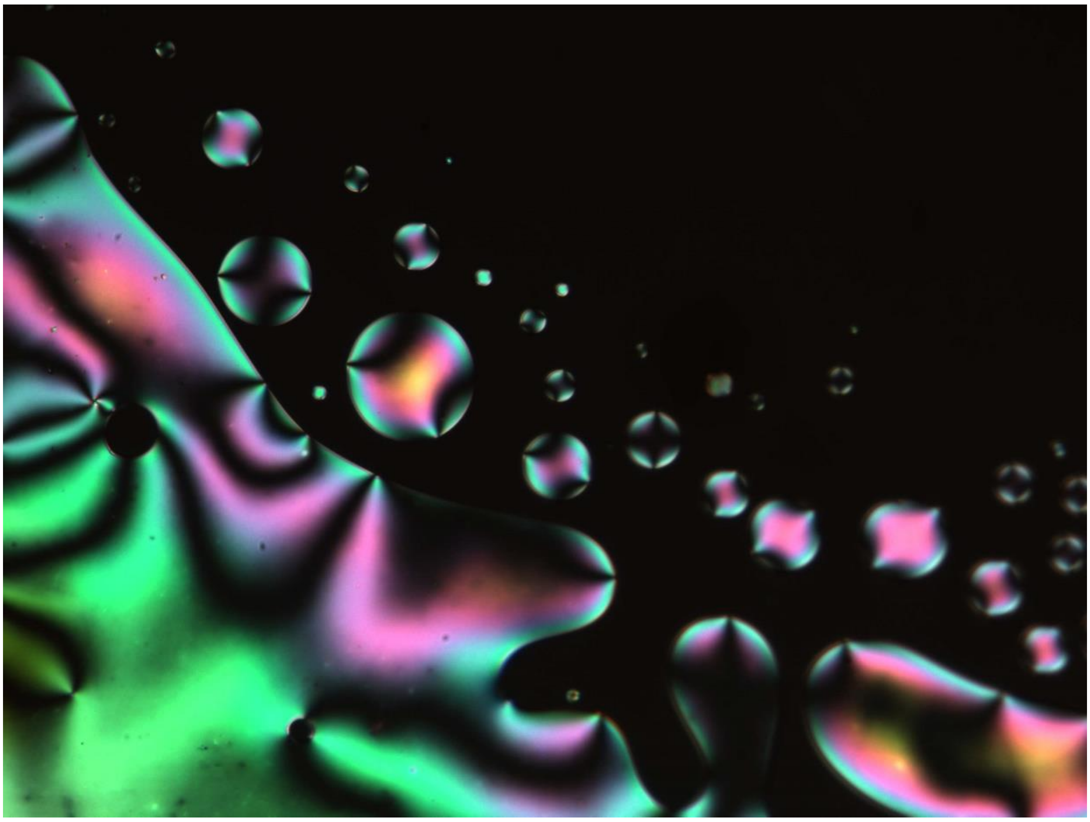

Figure S35. POM texture of the N-Iso transition of compound *33_D* (T = 186.1 °C).

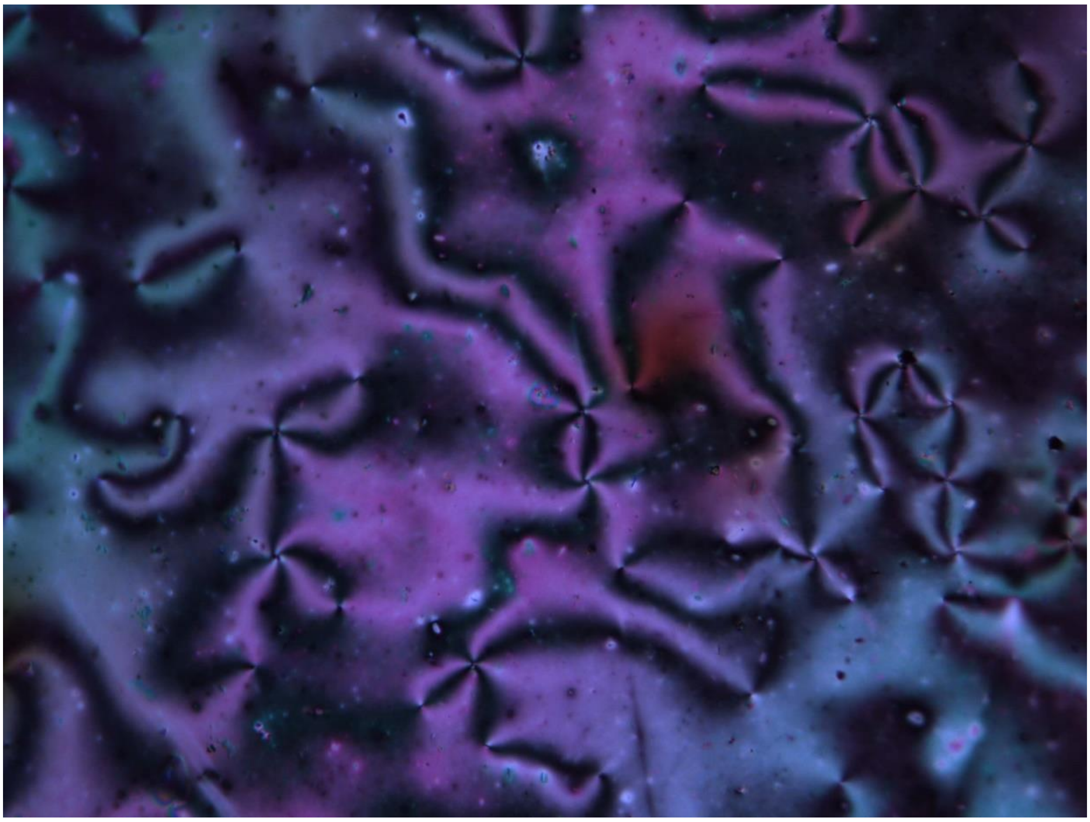

Figure S36. POM texture of nematic phase of compound *25_D* (T = 135.0 °C).

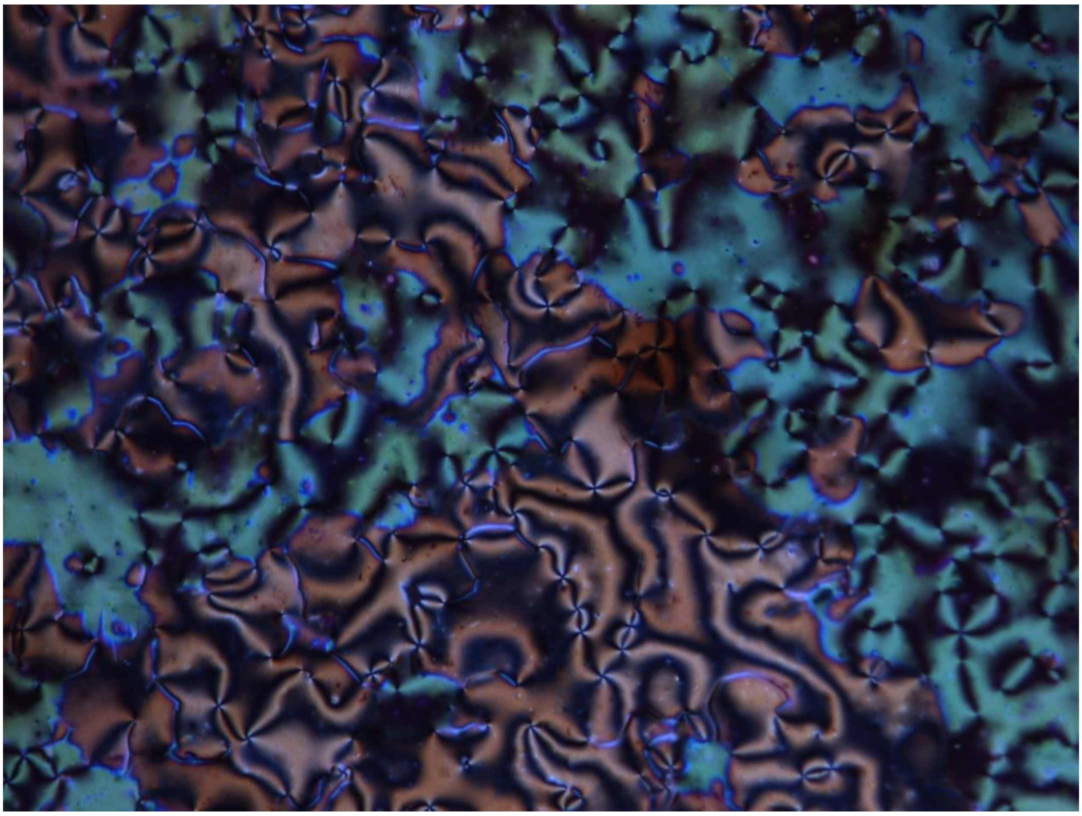

Figure S37. POM texture of nematic phase of compound *35_D* (T = 110.5 °C).
